# Association of Frailty and Frailty Trajectory with Risk for Respiratory Infectious Diseases

**DOI:** 10.3201/eid3206.251235

**Published:** 2026-06

**Authors:** Jin Yang, Hao Yan, Huan Chen, Dan Liu, Zhihao Li, Chen Mao

**Affiliations:** School of Public Health, Southern Medical University, Guangzhou, China; National Institute of Health Data Science of China, Southern Medical University, Guangzhou

**Keywords:** Respiratory infections, influenza, viruses, frailty, respiratory infectious diseases, physical frailty, frailty index, risk factors, China, United Kingdom

## Abstract

We explored the association between frailty and respiratory infectious diseases (RIDs) through a large cohort of 423,691 participants in the UK Biobank. Participants without baseline RIDs were assessed by physical frailty and frailty index. A total of 16,848 participants had repeated assessments. We divided participants into nonfrail, prefrail, and frail groups and categorized frailty changes as alleviation, maintenance, or aggravation. We estimated risk for RIDs, including influenza, pneumonia, and other acute lower respiratory infections. Compared with nonfrailty, prefrailty and frailty increased risk for RIDs 1.32–2.29 times. Each 0.1-point increase in frailty index per year raised risk for RIDs by 47%; each 1-point increase in physical frailty per year increased risk by 26%. Frailty worsening (e.g., aggravation of prefrailty) amplified risk by 2.31–4.16 times. Partial frailty improvement did not fully eliminate risk. Frailty is a modifiable, dynamic risk factor, underscoring the need for early frailty identification and intervention to reduce RIDs in high-risk populations.

Respiratory infectious diseases (RIDs) seriously affect human health (*1*,*2*). Before COVID-19, pneumonia and influenza were the primary fatal RIDs, particularly among the older adult population (*3*,*4*). Given the substantial burden of respiratory infections on health, identifying factors that contribute to reduced life expectancy related to RIDs is crucial for advancing public health.

Frailty is a clinical syndrome characterized by increased vulnerability caused by decreased physiologic reserves and functions of multiple physiologic systems (*5*,*6*). Two main approaches are used to assess frailty: the frailty phenotype and the deficit accumulation model (*7*,*8*). Growing evidence has demonstrated that frailty increases the risk for various adverse outcomes, including falls, Parkinson’s disease, and death (*9*–*11*). Recent reviews have further highlighted frailty as a notable clinical and public health issue in older adults (*12*). Studies also have linked frailty to chronic obstructive pulmonary disease, asthma, and infectious diseases (*13*–*16*). However, previous studies on the relationship between frailty and RID have been limited by insufficient sample sizes, and evidence regarding the impact of frailty changes over time remains inconclusive. To address those gaps, we examined the associations of frailty and its long-term changes with risk for RIDs by leveraging data from a large UK cohort.

## Methods

We conducted a population-based prospective cohort study by analyzing data from the UK Biobank (https://www.ukbiobank.ac.uk), which recruited ≈500,000 participants 37–73 years of age across England, Wales, and Scotland. Baseline data were collected during 2006–2010 through touchscreen questionnaires capturing self-reported characteristics, verbal interviews for medical history, standardized physical measurements, and biological sample collection. In addition, participant data were linked to electronic health records for longitudinal follow-up. 

We assessed frailty by using a physical frailty (PF) phenotype and a frailty index (FI), and categorized persons as nonfrail, prefrail, or frail. We also ascertained incident RIDs from linked health records. From an initial cohort of 502,376 participants, we excluded persons with missing data on frailty variables (n = 1,988), prevalent RIDs at baseline (n = 56, 977), missing follow-up or recruitment information (n = 10), or m missing covariates (n = 19,710), resulting in an analytical cohort of 423,691 participants for baseline analysis. In the longitudinal analysis of frailty changes, we included a subset of 16,848 participants who had complete follow-up frailty assessments and did not have interim outcomes (Appendix Figure 1).

### Assessment of PF and FI

We used the Fried Frailty Phenotype assessment (*7*) to determine PF. In brief, the Fried model assesses 5 criteria: 3 self-reported components (unintended weight loss, fatigue, and low physical activity) and 2 objectively measured components (slow walking speed and reduced grip strength). We divided participants into 3 groups according to the number of Fried model criteria met: nonfrail (0 criteria), prefrail (1–2 criteria), or frail (>3 criteria). 

We used the FI developed by researchers using a population of 500,000 participants from the UK Biobank (*17*). In brief, we calculated FI from a set of 49 self-reported items covering health, diseases and disabilities, and mental well-being, according to standard protocol. For participants with <10 missing responses, we computed FI as the ratio of reported health or functional problems to the total number of possible items, yielding a score of 0–1. Finally, we classified participants as nonfrail (FI <0.12), prefrail (FI 0.12–0.24), or frail (FI >0.24). We applied those classifications to both baseline and follow-up assessments of PF and the FI.

### Definitions of Outcomes

We defined study outcomes as the first occurrence of RIDs as classified in the International Classification of Diseases, 10th Revision (ICD-10). The primary outcome was a composite of influenza (ICD-10 codes J09–J11), pneumonia (ICD-10 codes J12–J18), and other acute lower respiratory tract infections (OALRTI; ICD-10 codes J20–J22). We also analyzed those conditions individually as secondary outcomes. We determined study outcomes by linking National Health Service (NHS) records, including hospital inpatient data and death registry records. Detailed information about the linkage process is available on UK Biobank.

### Covariables

We considered the following potential confounders: age; sex (male or female); ethnicity (White or another race); education level (high, intermediate, or low); Townsend deprivation index (TDI); smoking status (never, former, or current); alcohol consumption status (never, former, or current); cumulative dietary risk factor score (continuous, ranging from 0 to 9 [most healthy to least healthy]); body mass index (BMI, kg/m^2^) category (underweight BMI <18.5, normal weight BMI 18.5 to <25, overweight BMI 25 to <30, or obese BMI >30); sleep duration (continuous, hours/day); sleeplessness (never, sometimes, or usually); and daytime dozing (never, sometimes, or often). We also examined cofounders related to exposure to pollution, including exposure to particulate matter with an aerodynamic diameter of <2.5 µm (PM_2.5_ exposure; continuous, μg/m^3^), nitrogen dioxide (continuous, μg/m^3^), and nitrogen oxide (continuous, μg/m^3^) (Appendix Table 1).

### Statistical Analysis

We calculated baseline and follow-up characteristics as mean (SD) or median (IQR) for continuous variables and as no. (%) for categorical variables, which we stratified by frailty status (nonfrail, prefrail, and frail). We used analysis of variance (ANOVA) or Kruskal-Wallis H test to compare differences for continuous variables and χ^2^ test to compare categorical variables. The primary outcome was any first-recorded RIDs (composite of influenza, OALRTI, and pneumonia); we further analyzed each of the 3 constituent RIDs as a secondary outcome. We calculated time-to-event from the baseline or first follow-up date until the first RID event, death, or December 31, 2022, whichever occurred first. We used Kaplan-Meier curves to estimate cumulative incidence and compared results by using log-rank tests. We used Cox proportional hazards models to estimate the hazard ratio (HR) and 95% CI for the risk of RIDs and each secondary outcome. 

We analyzed frailty both as a categorical variable and as a continuous variable using a 1-unit increment for PF and a 0.1-unit increment for FI score. Fully adjusted models further incorporated restricted cubic splines to evaluate potential nonlinear associations between sustained frailty and risk of RIDs. We considered and adjusted for potential confounders in multivariable models. Model 1 adjusted for age and sex, and model 2 further adjusted for full covariables.

### Frailty Trajectories

We derived frailty trajectories by fitting a linear regression model for each participant and using the PF (range 0–5) or FI (range 0–1) score as the dependent variable and follow-up time as the independent variable. We calculated change in FI (ΔFI) or PF (ΔPF) in points per year, which represented a person’s frailty trajectory. We defined the association between baseline and follow-up changes in frailty status and RIDs risk. We categorized long-term changes in frailty status as alleviation, maintenance, or aggravation, then included 7 study categories: nonfrailty maintenance, nonfrailty aggravation, prefrailty alleviation, prefrailty maintenance, prefrailty aggravation, frailty alleviation, and frailty maintenance. We used nonfrailty maintenance as the reference group. For ΔFI and ΔPF, values >0 indicated that the degree of frailty increased over time, a value of 0 indicated stable degree of frailty, and values <0 indicated improvement in degree of frailty. Further, we calculated the population attributable risk for RIDs events to estimate the population-level risk associated with changes in frailty status.

### Subgroup Analyses

For analyses, we stratified subgroups by age (<65 vs. >65 years) and sex. To address potential reverse causality, we performed a sensitivity analysis by excluding events occurring within the first 2 years of follow-up. Another sensitivity analysis excluded data after 2020 to mitigate the confounding effects of the COVID-19 pandemic. We further evaluated the association between frailty and cause-specific RIDs risk by using Fine-Gray competing risks regression models and treating all-cause mortality as a competing event (Appendix Table 2). We conducted 2-tailed analyses in R version 4.3.1 (The R Project for Statistical Computing, https://www.r-project.org) and considered p<0.05 statistically significant.

## Results

### Baseline Characteristics

The first assessment cohort included 423,691 persons with a mean age of 56.35 years; 45.5% were male and 54.5% were female (Table 1). In that assessment, we classified 2.14% as frail and 34.16% prefrail over a mean follow-up period of 13.79 years (Table 1). The final assessment subcohort had a mean follow-up of 9.58 years and included 16,848 persons, 2.43% of whom were classified as frail and 49.56% as prefrail. Persons classified as frail were generally older, were more likely to be female, and had lower education levels. Frail persons also had higher prevalences of smoking, socioeconomic deprivation, adverse sleep patterns, poorer diet quality, and higher air pollution exposure and were more likely to be overweight or obese. Consistent with their risk profiles, frail participants had a markedly higher incidence of RIDs than nonfrail participants.

**Table 1:** Baseline characteristics of participants in the first and final assessment of physical frailty in a study of association of frailty and frailty trajectory with risk for RIDs*

Characteristics	First assessment					Final assessment			
	Frail, n = 9,075	Prefrail, n = 144,734	Nonfrail, n = 269,882	p value†		Frail, n = 409	Prefrail, n = 8,350	Nonfrail, n = 8,089	p value†
Mean follow-up, y (SD)	12.29 (3.34)	13.02 (2.64)	13.37 (2.25)	<0.001		8.96 (2.33)	9.50 (1.59)	9.69 (1.44)	<0.001
Mean age, y (SD)	57.86 (7.63)	56.96 (8.08)	55.97 (8.09)	<0.001		62.40 (7.52)	62.10 (7.29)	60.36 (7.46)	<0.001
Sex				<0.001					<0.001
M	3,327 (36.7)	62,077 (42.9)	127,389 (47.2)			169 (41.3)	3,950 (47.3)	4,144 (51.2)	
F	5,748 (63.3)	82,657 (57.1)	142,493 (52.8)			240 (58.7)	4,400 (52.7)	3,945 (48.8)	
Ethnicity				<0.001					<0.001
White	7,793 (85.9)	133,415 (92.2)	259,028 (96.0)			389 (95.1)	8,122 (97.3)	7,927 (98.0)	
Another race	1,282 (14.1)	11,319 (7.8)	10,854 (4.0)			20 (4.9)	228 (2.7)	162 (2.0)	
Education				<0.001					<0.001
High	1,398 (15.4)	41,353 (28.6)	99,556 (36.9)			106 (25.9)	3,505 (42.0)	3,868 (47.8)	
Intermediate	4,061 (44.7)	73,466 (50.8)	135,111 (50.1)			219 (53.5)	4,056 (48.6)	3,688 (45.6)	
Low	3,616 (39.8)	29,915 (20.7)	35,215 (13.0)			84 (20.5)	789 (9.4)	533 (6.6)	
Smoking status				<0.001					<0.001
Never	4,229 (46.6)	76,799 (53.1)	154,029 (57.1)			209 (51.1)	4,909 (58.8)	4,944 (61.1)	
Previous	3,047 (33.6)	50,681 (35.0)	91,828 (34.0)			156 (38.1)	2,918 (34.9)	2,676 (33.1)	
Current	1,799 (19.8)	17,254 (11.9)	24,025 (8.9)			44 (10.8)	523 (6.3)	469 (5.8)	
Alcohol status				<0.001					<0.001
Never	1,060 (11.7)	8,211 (5.7)	8,440 (3.1)			30 (7.3)	320 (3.8)	220 (2.7)	
Previous	1,025 (11.3)	6,629 (4.6)	6,649 (2.5)			37 (9.0)	275 (3.3)	184 (2.3)	
Current	6,990 (77.0)	129,894 (89.7)	254,793 (94.4)			342 (83.6)	7,755 (92.9)	7,684 (95.0)	
Mean (SD) Townsend deprivation index	0.74 (3.59)	−0.93 (3.24)	−1.66 (2.88)	<0.001		−0.54 (3.44)	−1.91 (2.75)	−2.27 (2.53)	<0.001
Mean (SD) sleep duration, h/d	6.81 (2.22)	7.07 (1.37)	7.15 (1.08)	<0.001		6.97 (2.19)	7.22 (1.16)	7.21 (0.99)	<0.001
Mean (SD) cumulative dietary risk factor score	5.29 (1.45)	4.99 (1.42)	4.91 (1.38)	<0.001		5.29 (1.38)	4.88 (1.33)	4.87 (1.32)	<0.001
Mean (SD) PM_2.5_, μg/m^3^	10.34 (1.11)	10.07 (1.06)	9.93 (1.04)	<0.001		10.26 (1.13)	10.01 (1.06)	9.92 (1.02)	<0.001
Mean (SD) NO_2_, μg/m^3^	29.28 (7.69)	27.33 (7.64)	26.23 (7.56)	<0.001		28.02 (6.97)	26.35 (6.92)	25.71 (6.72)	<0.001
Mean (SD) NO, μg/m^3^	48.91 (16.56)	45.24 (15.86)	43.19 (15.28)	<0.001		47.05 (14.94)	43.78 (14.63)	42.46 (13.87)	<0.001
BMI category‡				<0.001					<0.001
Underweight	62 (0.7)	758 (0.5)	1,184 (0.4)			3 (0.7)	36 (0.4)	46 (0.6)	
Normal weight	1,369 (15.1)	36,742 (25.4)	100,907 (37.4)			68 (16.8)	2,577 (31.1)	3,481 (43.6)	
Overweight	2,728 (30.1)	60,162 (41.6)	118,895 (44.1)			131 (32.4)	3,664 (44.2)	3,369 (42.2)	
Obese	4,916 (54.2)	47,072 (32.5)	48,896 (18.1)			202 (50.0)	2,013 (24.3)	1,096 (13.7)	
RIDs				<0.001					<0.001
N	6,882 (75.8)	125,900 (87.0)	246,801 (91.4)			337 (82.4)	7,695 (92.2)	7,627 (94.3)	
Y	2,193 (24.2)	18,834 (13.0)	23,081 (8.6)			72 (17.6)	655 (7.8)	462 (5.7)	

*Values are no. (%) except as indicated. Data are from UK Biobank (https://www.ukbiobank.ac.uk) during 2006–2010 for the first assessment and during 2012–2013 for the final assessment. BMI, body mass index; PM_2.5_, particulate matter <2.5 mm; NO, nitrogen oxide; NO_2_, nitrogen dioxide; RIDs, respiratory infectious diseases. †Calculated by analysis of variance, Kruskal-Wallis H, or χ^2^ test. p<0.05 considered statistically significant. ‡Underweight <18.5 kg/m^2^; normal weight, 18.5 kg/m^2^ to <25 kg/m^2^; overweight, 25 kg/m^2^ to <30 kg/m^2^; obese, >30 kg/m^2^.

Overall, baseline patterns for FI were broadly consistent with those observed using PF, and we noted substantial concordance between the 2 measures in both the first and final assessments (Appendix Table 3, 4). The distribution of respiratory infection subtypes was similar across frailty groups, regardless of the assessment tool or time point (Appendix Figure 2). A comparison of participants with and without follow-up data revealed statistically significant but clinically modest differences, suggesting a low risk for meaningful selection bias (Appendix Table 5).

### Incident RIDs HRs Associated with Frailty

During a mean follow-up of 13.79 years in the first assessment, a total of 44,108 cases of incident RIDs were recorded. In fully adjusted models, both frailty measures showed a dose-dependent increase in RID risk. For PF, adjusted HR for the prefrail group was 1.32 (95% CI 1.30–1.35) and for the frail group adjusted HR was 2.01 (95% CI 1.92–2.10). For FI, the corresponding HR for the prefrail group was 1.50 (95% CI 1.47–1.53) and for the frail group was 2.29 (95% CI 2.22–2.37) (Table 2).

**Table 2:** Hazard ratios of incident RIDs in first assessment of association of frailty and frailty trajectory with risk for RIDs*

Category	Events/no. participants	Model 1			Model 2	
		HR (95% CI)	p value		HR (95% CI)	p value
Grouped by physical frailty						
Nonfrail	23,081/269,882	Referent			Referent	
Prefrail	18,834/144,734	1.51 (1.48–1.54)	<0.001		1.32 (1.30–1.35)	<0.001
Frail	2,193/9,075	2.93 (2.81–3.06)	<0.001		2.01 (1.92–2.10)	<0.001
Per 1-point increase		1.41 (1.39–1.42)	<0.001		1.26 (1.24–1.27)	<0.001
Grouped by frailty index						
Nonfrail	19,181/253,296	Referent			Referent	
Prefrail	19,014/144,591	1.68 (1.65–1.71)	<0.001		1.50 (1.47–1.53)	<0.001
Frail	5,913/25,804	3.02 (2.93–3.11)	<0.001		2.29 (2.22–2.37)	<0.001
Per 0.1-point increase		1.64 (1.62–1.66)	<0.001		1.47 (1.45–1.49)	<0.001
RID subgroups						
Influenza						
Grouped by physical frailty						
Nonfrail	1,209/269,882	Referent			Referent	
Prefrail	931/144,734	1.42 (1.30–1.55)	<0.001		1.30 (1.19–1.42)	<0.001
Frail	132/9,075	3.18 (2.65–3.80)	<0.001		2.44 (2.02–2.95)	<0.001
Per 1-point increase		1.39 (1.33–1.46)	<0.001		1.29 (1.23–1.36)	<0.001
Grouped by frailty index						
Nonfrail	1,054/253,296	Referent			Referent	
Prefrail	924/144,591	1.51 (1.38–1.65)	<0.001		1.41 (1.29–1.54)	<0.001
Frail	291/25,804	2.64 (2.31–3.00)	<0.001		2.19 (1.90–2.51)	<0.001
Per 0.1-point increase		1.52 (1.44–1.59)	<0.001		1.41 (1.34–1.49)	<0.001
OALRTI						
Grouped by physical frailty						
Nonfrail	13,070/269,882	Referent			Referent	
Prefrail	10,308/144,734	1.44 (1.40–1.48)	<0.001		1.27 (1.24–1.30)	<0.001
Frail	1,131/9,075	2.52 (2.37–2.68)	<0.001		1.78 (1.67–1.90)	<0.001
Per 1-point increase		1.34 (1.33–1.36)	<0.001		1.21 (1.19–1.23)	<0.001
Grouped by frailty index						
Nonfrail	10,595/253,296	Referent			Referent	
Prefrail	10,677/144,591	1.70 (1.66–1.75)	<0.001		1.54 (1.49–1.58)	<0.001
Frail	3,237/25,804	2.89 (2.78–3.01)	<0.001		2.26 (2.17–2.36)	<0.001
Per 0.1-point increase		1.62 (1.59–1.64)	<0.001		1.47 (1.44–1.49)	<0.001
Pneumonia						
Grouped by physical frailty						
Nonfrail	11,221/269,882	Referent			Referent	
Prefrail	10,204/144,734	1.66 (1.62–1.71)	<0.001		1.43 (1.39–1.47)	<0.001
Frail	1,370/9,075	3.70 (3.50–3.92)	<0.001		2.39 (2.25–2.53)	<0.001
Per 1-point increase		1.52 (1.50–1.54)	<0.001		1.34 (1.32–1.36)	<0.001
Grouped by frailty index						
Nonfrail	9,413/253,296	Referent			Referent	
Prefrail	9,940/144,591	1.73 (1.68–1.78)	<0.001		1.52 (1.47–1.56)	<0.001
Frail	3,442/25,804	3.39 (3.26–3.53)	<0.001		2.45 (2.35–2.56)	<0.001
Per 0.1-point increase		1.73 (1.70–1.76)	<0.001		1.51 (1.49–1.54)	<0.001

*Data from UK Biobank (https://www.ukbiobank.ac.uk) collected during 2006–2010. Model 1 is adjusted for age and sex; model 2 is adjusted for all covariates. HRs and 95% CIs were estimated using Cox proportional hazards models. A 2-sided p<0.05 was considered statistically significant. FI, frailty index; HR, hazard ratio; OALRTI, other acute lower respiratory tract infection; PF, physical frailty; RID, respiratory infectious disease.

We observed similar trends in the final assessment cohort. Mean follow-up was 9.58 years, during which 1,189 incident RIDs cases were reported. PF-based HR was 1.17 (95% CI 1.04–1.33) for prefrail group and 2.27 (95% CI 1.75–2.95) for the frail group. FI-based HR was 1.28 (95% CI 1.13–1.45) for the prefrail group and 2.59 (95% CI 2.10–3.19) for the frail group (Table 3). Kaplan-Meier and restricted cubic spline analyses further supported those associations (Appendix Figures 3–6).

**Table 3:** Hazard ratios of incident RIDs in final assessment of association of frailty and frailty trajectory with risk for RIDs*

Category	Events/no. participants	Model 1			Model 2	
		HR (95% CI)	p value		HR (95% CI)	p value
Grouped by physical frailty						
Nonfrail	462/8,089	Referent			Referent	
Prefrail	655/8,350	1.28 (1.14–1.45)	<0.001		1.17 (1.04–1.33)	0.011
Frail	72/409	3.04 (2.37–3.90)	<0.001		2.27 (1.75–2.95)	<0.001
Per 1-point increase		1.36 (1.27–1.46)	<0.001		1.25 (1.17–1.35)	<0.001
Grouped by frailty index						
Nonfrail	573/10,424	Referent			Referent	
Prefrail	495/5,794	1.44 (1.27–1.62)	<0.001		1.28 (1.13–1.45)	<0.001
Frail	121/630	3.31 (2.72–4.03)	<0.001		2.59 (2.10–3.19)	<0.001
Per 0.1-point increase		1.66 (1.53–1.80)	<0.001		1.48 (1.36–1.62)	<0.001
RID subgroups						
Influenza						
Grouped by physical frailty						
Nonfrail	9/8,089	Referent			Referent	
Prefrail	20/8,350	2.04 (0.92–4.50)	0.078		1.81 (0.81–4.05)	0.146
Frail	2/409	4.04 (0.87–18.81)	0.075		2.36 (0.45–12.47)	0.312
Per 1-point increase		1.38 (0.96–1.98)	0.079		1.20 (0.79–1.80)	0.382
Grouped by frailty index						
Nonfrail	18/10,424	Referent			Referent	
Prefrail	9/5,794	0.85 (0.38–1.91)	0.696		0.73 (0.32–1.65)	0.443
Frail	4/630	3.35 (1.12–9.98)	<0.05		2.09 (0.63–6.95)	0.228
Per 0.1-point increase		1.82 (1.15–2.90)	<0.05		1.53 (0.94–2.50)	0.087
OALRTI						
Grouped by physical frailty						
Nonfrail	242/8,089	Referent			Referent	
Prefrail	301/8,350	1.12 (0.94–1.33)	0.194		1.03 (0.86–1.22)	0.760
Frail	28/409	2.13 (1.44–3.16)	<0.001		1.64 (1.09–2.48)	<0.05
Per 1-point increase		1.22 (1.10–1.36)	<0.001		1.14 (1.02–1.27)	0.024
Grouped by frailty index						
Nonfrail	278/10,424	Referent			Referent	
Prefrail	233/5,794	1.40 (1.18–1.67)	<0.001		1.28 (1.07–1.53)	0.007
Frail	60/630	3.29 (2.49–4.36)	<0.001		2.77 (2.06–3.73)	<0.001
Per 0.1-point increase		1.63 (1.45–1.83)	<0.001		1.51 (1.32–1.71)	<0.001
Pneumonia						
Grouped by physical frailty						
Nonfrail	247/8,089	Referent			Referent	
Prefrail	408/8,350	1.47 (1.26–1.73)	<0.001		1.34 (1.14–1.58)	<0.001
Frail	53/409	4.12 (3.06–5.55)	<0.001		2.96 (2.16–4.05)	<0.001
Per 1-point increase		1.53 (1.40–1.67)	<0.001		1.39 (1.27–1.52)	<0.001
Grouped by frailty index						
Nonfrail	320/10,424	Referent			Referent	
Prefrail	312/5,794	1.58 (1.35–1.84)	<0.001		1.38 (1.17–1.62)	<0.001
Frail	76/630	3.53 (2.75–4.55)	<0.001		2.67 (2.04–3.50)	<0.001
Per 0.1-point increase		1.73 (1.56–1.92)	<0.001		1.52 (1.35–1.70)	<0.001

*Data from UK Biobank (https://www.ukbiobank.ac.uk) collected during 2012–2013. Model 1 is adjusted for age and sex; model 2 is adjusted for all covariates. HRs and 95% CIs were estimated using Cox proportional hazards models. A 2-sided p<0.05 was considered statistically significant. FI, frailty index; HR, hazard ratio; OALRTI, other acute lower respiratory tract infection; PF, physical frailty; RID, respiratory infectious disease.

### Associations of Frailty Trajectory with RIDs

**Figure 1:**
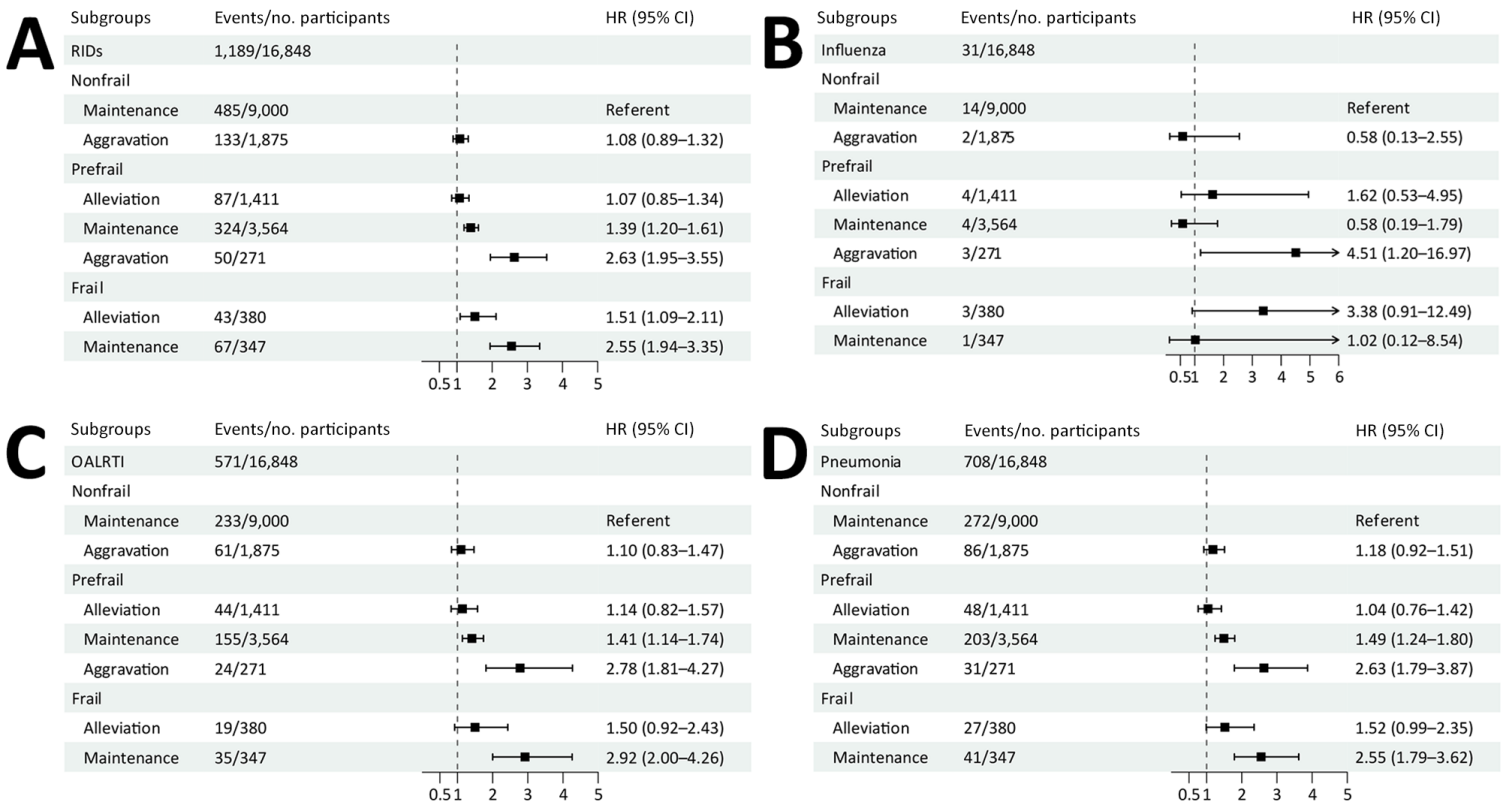
Longitudinal baseline and follow-up associations of frailty index with risk for RIDs. Forest plots show the trajectory of frailty index associated with incident RIDs (A), influenza (B), OALRTI (C), and pneumonia (D). Squares indicate point estimates of HRs; error bars indicate 95% CI; dotted vertical lines indicate null effect. Analyses were adjusted for age, sex, ethnicity, education level, Townsend deprivation index, smoking status, alcohol consumption status, cumulative dietary risk factor score, PM_2.5_ (exposure to particulate matter <2.5 μm), nitrogen dioxide and nitrogen oxide levels, body mass index category, sleep duration, sleeplessness, and daytime dozing. HR, hazard ratio; OALRTI, other acute lower respiratory tract infection; RIDs, respiratory infectious diseases.

In the analysis of associations between FI trajectories and RIDs, we noted substantially higher risks among the maintained prefrailty (HR 1.39 [95% CI 1.20–1.61]), aggravation of prefrailty (HR 2.63 [1.95–3.55]), alleviation of frailty (HR 1.51 [1.09–2.11]), and maintained frailty (HR 2.55 [1.94–3.35]) subgroups. We also observed similar increases in risk in OALRTI and pneumonia, particularly in the aggravation of prefrailty and maintained frailty groups, where the risk increased by 2.55–2.92 times (Figure 1).

**Figure 2:**
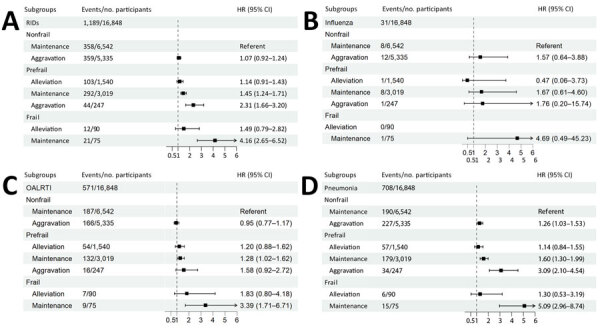
Longitudinal baseline and follow-up associations of physical frailty with risk for RIDs. Forest plots show the trajectory of physical frailty associated with incident RIDs (A), influenza (B), OALRTI (C), and pneumonia (D). Squares indicate point estimates of HRs; error bars indicate 95% CI; dotted vertical lines indicate null effect. Analyses were adjusted for age, sex, ethnicity, education level, Townsend deprivation index, smoking status, alcohol consumption status, cumulative dietary risk factor score, PM_2.5_ (exposure to particulate matter <2.5 μm), nitrogen dioxide and nitrogen oxide levels, body mass index category, sleep duration, sleeplessness, and daytime dozing. HR, hazard ratio; OALRTI, other acute lower respiratory tract infection; RIDs, respiratory infectious diseases.

In the analysis of associations between PF trajectories and RIDs, we designated nonfrail as the reference group. For RIDs, we noted substantial increases in risk for the maintained prefrailty (HR 1.45 [95% CI 1.24–1.71]), aggravation of prefrailty (HR 2.31 [1.66–3.20]), and maintained frailty (HR 4.16 [2.65–6.52]) subgroups. We observed similar patterns for OALRTI and pneumonia (Figure 2).

**Figure 3:**
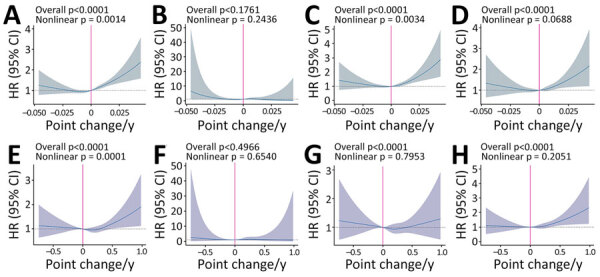
Restricted cubic spline curves from linear regression analysis of frailty changes associated with risk for respiratory infectious diseases. A–D) Dose-response relationships of change in frailty index associated with incident respiratory infectious diseases (A), influenza (B), other acute lower respiratory tract infection (C), and pneumonia (D). E–H) Dose-response relationships of change in physical frailty associated with incident respiratory infectious diseases (E), influenza (F), other acute lower respiratory tract infection (G), and pneumonia (H). Blue lines indicate fitted HR curves; shading indicates 95% CI; horizontal dotted lines indicate HR of 1; vertical lines indicate 0 change. Analyses were adjusted for age, sex, ethnicity, education level, Townsend deprivation index, smoking status, alcohol consumption status, cumulative dietary risk factor score, PM_2.5_ (exposure to particulate matter <2.5 μm), nitrogen dioxide and nitrogen oxide levels, body mass index category, sleep duration, sleeplessness, and daytime dozing. HR, hazard ratio.

Restricted cubic spline curves showed a statistically significant U-shaped relationship between frailty changes and RIDs risk (overall p<0.0001, nonlinearity p<0.01). Specifically, persons with deteriorating frailty trajectories (ΔFI or ΔPF >0) exhibited statistically significant and sharp increases in RIDs risk compared with those with stable frailty status (ΔFI or ΔPF = 0) (Figure 3).

### Additional Analyses

We noted similar effects in subgroup analyses (Appendix Tables 6–9) as on the main results. Frailty showed a stronger association with the risk for incident RIDs in persons >65 years of age and in female populations. To avoid the possibility of reverse causation and effects of the COVID-19 pandemic on RIDs, we conducted sensitivity analyses that excluded cases with study outcomes <2 years of follow-up or cases that occurred after 2020 (Appendix Table 10). We also analyzed the relationship between frailty and deaths caused by RIDs in subgroups and noted slightly lower HR estimates in the competing risk model, but estimates remained robust (Appendix Table 11).

## Discussion

In this large prospective cohort study, both baseline frailty and worsening frailty trajectories were associated with higher risk for RIDs. Those findings support frailty as a dynamic marker of vulnerability for identifying populations at elevated risk for RIDs. 

Previous studies explored the relationship between frailty and some RIDs, proving that frailty was closely related to RIDs occurrences (*18*,*19*). For example, a recent systematic review and meta-analysis focused on patients >60 years of age with pneumonia found that frailty was common (incidence rate of 49%) and greatly increased risk for death (odds ratio [OR] 3.51) and readmission (*20*). However, that study used a single point in time for frailty assessment, without considering the dynamic changes in frailty status. A prospective cohort study exploring the relationship between influenza and frailty found no association between influenza and frailty phenotype (OR 0.50) (*21*). However, that study had a small (n = 1,135) sample size and included persons >50 (median 67.5) years of age from the community, which might have underestimated the long-term association between frailty and influenza risk. That underestimation could result from potential underdetection of influenza cases, particularly milder or asymptomatic cases among frailer persons who might not have sought testing. Another study found that frailty was a predictive factor for poor recovery in patients >65 years of age hospitalized with acute respiratory diseases (OR 0.70–0.75) (*22*), but it only used data from the 2011–12, 2012–13, and 2013–14 influenza seasons, which limited the generalizability of the conclusions. In contrast, using a large sample size, long-term follow-up, and multidimensional assessments (PF and FI), our study revealed the independent associations of frailty and its changing trajectory on the risk for RIDs. For participants stratified by FI, prefrail persons had 1.50 times higher risk for RIDs and frail groups had 2.29 times higher risk compared with the nonfrail group. In the analysis of associations between FI trajectories and RIDs, risks substantially increased in the maintained prefrailty (HR 1.39), aggravation of prefrailty (HR 2.63), alleviation of frailty (HR 1.51), and maintained frailty (HR 2.55) groups. The results grouped according to PF were similar. Our study not only validated the role of frailty as a static risk factor but also quantified the cumulative effect of its dynamic changes on infection.

Previous studies have relied mainly on single point-of-time assessments of degeneration, failing to capture the dynamic evolution of degeneration and its cumulative biologic effects. Persons can gradually fall into a state of frailty, which can lead to adverse health outcomes, including cardiovascular disease, depression, and all-cause deaths (*23*–*25*). However, few studies have examined the relationship between dynamic changes in frailty and RIDs. Our study integrates longitudinal frailty trajectories and demonstrates that the maintenance or aggravation of frailty status can independently predict the long-term risk for RIDs. 

Unlike previous research limited to static frailty assessments, our repeated measurements revealed dynamic frailty patterns. Those findings emphasize that aggravation or maintenance of frailty were key predictors of RIDs risk, and that RIDs risk increased with worsening frailty trajectories. In addition, we found a U-shaped association between the annual rate of frailty progression (ΔFI or ΔPF) and susceptibility to RIDs, indicating that worsening frailty rapidly increases the risk for RIDs. Furthermore, we noted a higher risk for RIDs among participants whose frailty metrics improved. 

Frailty is a multidimensional syndrome, in which a person’s overall health status might not fully recover to nonfrailty levels, and certain subclinical states (such as chronic inflammation or metabolic disorders) might persist, resulting in a continued high risk for infection. We found that for every 0.1-point increase in FI per year, the risk for RIDs increased by 47%, and for every 1-point increase in PF per year, the risk increased by 26%. When conducting subgroup analyses, we found that for every 0.1-point increase in FI per year, the risk for influenza increased by 41%, OALRTI by 47%, and pneumonia by 51%; for every 1-point increase in PF per year, the risk for influenza increased by 29%, OALRTI by 21%, and pneumonia by 34%. The final assessment of frailty also showed similar outcomes. Those findings emphasize the value of continuous monitoring of frailty, especially in prefrail populations.

Several FI components, such as low physical activity and fatigue, are potential independent risk factors for respiratory infection and illness severity. However, frailty should not be viewed as a simple repetition of conventional epidemiologic risk factors because it reflects cumulative declines in physiologic reserve and multisystem dysregulation (*5*,*9*,*12*). In this study, we quantified vulnerability by using FI, which summarizes multidimensional health deficits; moreover, frailty is considered a distinct clinical syndrome rather than a synonym for underlying conditions or disability (*5*,*7*). Even after adjusting for major confounders including age, sex, education, deprivation, smoking, alcohol use, BMI, diet, sleep characteristics, and air pollution exposure, the association between frailty and RIDs remained evident, suggesting that frailty could capture integrated susceptibility beyond individual risk factors considered separately. At the same time, because some frailty components overlap with established infection-related risk factors, the independent causal contribution of frailty itself could not be fully disentangled in this observational study.

Our research also found that participants >65 years of age have a higher risk for developing RIDs because of prefrailty and frailty compared with persons <65 years of age. Results of a previous study emphasized that frailty affects the susceptibility to and severity of pneumonia in persons >65 years of age (*26*), which is similar to our results. 

Mounting evidence suggests that acute influenza infection could have long-lasting health effects, and studies have shown that frailty is associated with poor recovery after hospitalization for influenza (*22*,*27*). Our study also showed that frailty increases the risk of developing influenza by 119%–144% compared with nonfrailty. Those findings further support prioritizing vaccination-focused prevention among persons with frailty, particularly in resource-constrained settings where preventive interventions must be targeted efficiently.

Previous research has shown that frailty is associated with an increased risk for severe COVID-19 outcomes, including hospitalization or death (*28*). Our study extends those findings by showing that frailty was associated with a 1.29–2.20-fold increased risk for death from broader categories of RIDs. 

Frailty is associated with an exacerbated inflammatory state, which can impair immune function and increase susceptibility to respiratory infections (*29*). In addition, persons with frailty might be more likely to experience malnutrition, swallowing difficulties, and other underlying conditions (*26*), which could contribute to their susceptibility to RIDs and support the need for nutritional and rehabilitation-based intervention as targets for future study. 

Although some factors associated with frailty, such as smoking and alcohol use, can be modified at the patient level, frailty is also shaped by broader social and environmental determinants, including socioeconomic disadvantage and environmental exposures (*9*,*12*). Thus, frailty should not be interpreted as a condition that can be readily prevented through patient-level behavioral advice alone. Accordingly, future prevention efforts should consider broader public health and social policy measures, such as strengthening nutritional support and reducing exposure to harmful environments, and specific measures would depend on local resources and policy conditions. 

From a public health perspective, our findings demonstrate that vulnerable populations are at high risk for respiratory infections, and vaccination should be considered for this population. Furthermore, measures such as nutritional support and physical rehabilitation could be potential intervention targets, and those approaches might be better suited for exploration in select high-risk subgroups or demonstration projects. In that sense, our findings are intended not to advocate the immediate broad rollout of all frailty-directed interventions but to provide an epidemiologic basis for risk stratification so that preventive resources, including vaccination and other supportive strategies, can be more rationally prioritized.

The strengths of this study included a prospective design, a large sample size, dual-dimensional frailty assessment using PF and FI, and dynamic change analysis. The first limitation of this study is that unmeasured variables, such as genetic susceptibility or undiagnosed underlying conditions, might have led to residual confounding. Second, the baseline age range of the cohort was 37–73 years, and most participants were White, which limited the generalizability of the results to the entire population. Third, recall bias might have affected some self-reported information, including parts of the PF assessment and all FI items. Fourth, outcomes on the basis of ICD coding might have underestimated mild or unreported RIDs outcomes. Fifth, the UK Biobank did not have records for influenza and pneumonia vaccine administration, making analysis between the role of vaccination in frailty and RIDs impossible. Sixth, our models did not incorporate season-specific effects, and relying on time to first respiratory infection as the primary outcome could underestimate the true frequency and cumulative burden of recurrent respiratory infections. Finally, although we analyzed the frailty trajectory through longitudinal modeling, the evaluation interval might not have captured short-term fluctuations in frailty status. Future research should incorporate more frequent measurements of frailty and explore the mediating biologic mechanisms between frailty and respiratory infections, such as chronic inflammation and immune dysregulation.

In summary, this study provides strong evidence that baseline and dynamic frailty substantially increase the risk for RIDs. Our findings advocate for the inclusion of frailty screening as a preventive strategy for respiratory infections, especially in persons >65 years of age.

Appendix Additional information on association of frailty and frailty trajectory with risk for respiratory infectious diseases.
