# Supplementary material for: Association of Frailty and Frailty Trajectory with Risk for Respiratory Infectious Diseases
Source: Emerg Infect Dis. 2026 Jun;32(6):894–904. doi: 10.3201/eid3206.251235 (PMC13245239; doi:10.3201/eid3206.251235)
Supplement: Appendix — Additional information on association of frailty and frailty trajectory with risk for respiratory infectious diseases. [file 25-1235-Techapp-s1.pdf]

Article DOI: https://doi.org/10.3201/eid3206.251235

EID cannot ensure accessibility for supplementary materials supplied by authors. Readers who have difficulty accessing supplementary content should contact the authors for assistance.

Association of Frailty and Frailty Trajectory with Risk for Respiratory Infectious Diseases

Appendix

Definitions and Assessment Methods for Covariates

1. Ethnicity

Ethnicity was categorized as White, including participants identifying as British, Irish, or any other White background, and another race, which included all non-White participants, including those who reported more than one race.

2. Education

Education level was classified as High (college or university degree), Intermediate (A levels, O levels/GCSEs, CSEs, NVQ/HND/HNC, or other professional qualifications such as nursing or teaching), and Low (no formal qualifications).

3. Townsend deprivation index (TDI)

The Townsend deprivation index is a composite measure of material deprivation derived from UK census data, representing relative socioeconomic deprivation at the neighborhood level. Higher TDI scores indicate greater levels of deprivation, with negative values representing less deprived areas and positive values representing more deprived areas. The index was assigned to participants based on their residential postcodes at the time of recruitment.

4. $PM_{2.5}$ exposure (continuous, μg/m^3)

Particulate matter with an aerodynamic diameter of 2.5 μm or less (PM2.5) exposure was assessed by the UK Biobank. Annual average concentrations were estimated for each participant's residential address at first assessment (2006–2010). PM2.5 levels were treated as a continuous variable (μg/m^3) in our analyses, representing long-term ambient exposure to fine particulate matter.

5. NO_2 exposure (continuous, μg/m^3)

NO2 concentrations were analyzed as a continuous variable (μg/m^3), reflecting exposure to this traffic-related air pollutant.

6. Nitrogen oxides exposure (continuous, μg/m^3)

Nitrogen oxides (NOx) serving as a broader indicator of combustion-related air pollution, particularly from traffic sources. Like other air pollutants, NOx exposure was treated as a continuous variable (μg/m^3) in our analyses, based on annual average concentrations at baseline.

7. Cumulative dietary risk factor score

To assess the confounding factors in individuals' dietary behaviors, our study constructs the cumulative dietary risk factor score. The score is based on nine important dietary factors: fruit and vegetable intake, total fish intake, processed meat intake, red meat intake, milk type used, spread type, cereal intake, salt added to food, and water intake. Each factor is quantified according to specific scoring criteria, reflecting the healthiness of the dietary habit, and a total score is calculated by summing the individual scores. The score ranges from 0 to 9, with 0 representing the healthiest dietary habits and 9 representing the least healthy (Appendix Table 1).

Appendix Table 1. Cumulative dietary risk factors definitions used in the UK Biobank study (UK, 2006–2010 and 2012–2013)

Variable	Diet component	Field IDs	Unit conversion	Amount per serving
Fruit & vegetables				0 ≥5 serving/day (Ref.)
	Fresh fruit intake (pieces/day)	1309	Amount per serving: 1 piece	
	Dried fruit intake (pieces/day)	1319	Amount per serving: 2 piece	
	Cooked vegetable intake	1289	Amount per serving: 2 heaped	
	Salad/raw vegetable intake	1299	Amount per serving: 2 heaped	
Total fish intake				0 ≥2 times a week (at
	Oily fish intake (per week)	1329	0.5 Less than one	
	Non-oily fish intake (per	1339	0.5 Less than one	
Processed meat				0 ≤Once a week (Ref.)
	Processed meat intake (per	1349	0.5 Less than one	
Red meat				0 ≤Once a week (Ref.)
	Beef intake (per week)	1369	0.5 Less than one	
	Lamb/mutton intake (per	1379	0.5 Less than one	
	Pork intake (per week)	1389	0.5 Less than one	
Milk type used	Milk type used	1418		0 Semi-
Spread type	Spread type	1428		0 Never/rarely (Ref.)
Cereal intake	Cereal intake (Bowls/week)	1458	Amount per serving: Bran/oat/muesli	0 >5 bowls (Ref.)
Salt added to food	Salt added to food	1478		0 Never/rarely (Ref.)
Water intake	Water intake (Glasses/day)	1528		0 ≥6 glasses (Ref.)

*Field IDs and serving sizes used per diet component in UK Biobank with available data from the general baseline questionnaire. If participants achieved the intake goal they were considered to have an adequate intake of the diet component. Adequate intake of at least half of all diet components was considered as an ideal diet, less than half was considered a poor diet.

Appendix Table 2. Overview of statistical models and key analyses

Statistical models	Purpose		Exposures	Outcomes	Covariates	Sample Size	Results
Descriptive Statistics	RIDs risk by frailty status at first and final assessments	Compare baseline characteristics across frailty groups	Frailty status (non-frail, pre-frail, frail)	-	-	First assessment (n = 423,691)/ Final assessment (n = 16,848)/ First assessment-only(n = 406,843)	Table 1; Appendix Tables 3, 5
Kaplan-Meier Curves and Log-Rank Test		Plot and compare cumulative incidence of RIDs over time by frailty status	Frailty status	RIDs (influenza, OA-LRTI, pneumonia)	-	First assessment (n = 423,691)/ Final assessment (n = 16,848)	Appendix Figure 3; Appendix Figure 4
Cox Proportional Hazards Models (COX)		Estimate risk of RIDs associated with frailty groups and quantify frailty levels	Frailty status/ Continuous frailty (1-unit PF, 0.1-unit FI)	RIDs (influenza, OA-LRTI, pneumonia)	age, sex in model 1; Full covariates in model 2	First assessment (n = 423,691)/ Final assessment (n = 16,848)	Tables 2, 3
Restricted Cubic Splines (RCS)		Assess nonlinear dose-response associations between continuous frailty and incident RIDs	Continuous frailty (1-unit PF, 0.1-unit FI)	RIDs (influenza, OA-LRTI, pneumonia)	Full covariates	First assessment (n = 423,691)/ Final assessment (n = 16,848)	Appendix Figure 5; Appendix Figure 6
Linear regression	Frailty trajectory and RIDs	Derive individual frailty trajectory as a continuous measure over time (ΔFI, ΔPF)	Follow-up time (years)	Frailty score (PF 0–5; FI 0–1)	-	Final assessment (n = 16,848)	-
COX		Estimate risk of RIDs associated with long-term changes in frailty status (seven status)	Long-term changes in frailty status	RIDs (influenza, OA-LRTI, pneumonia)	Full covariates	Final assessment (n = 16,848)	Figures 1, 2

Statistical models	Purpose		Exposures	Outcomes	Covariates	Sample Size	Results
RCS		Assess nonlinear dose-response associations between frailty change (ΔFI/ΔPF) and incident	ΔFI/ΔPF	RIDs (influenza, OA-LRTI, pneumonia)	Full covariates	Final assessment (n = 16,848)	Figure 3
Subgroup Analyses (Stratified COX)	Subgroup and Sensitivity Analyses	Examine RIDs risk by age and sex	Frailty status/Continuous frailty/Long-term changes in frailty status	RIDs (influenza, OA-LRTI, pneumonia)	age, sex in model 1; Full covariates in model 2	Stratified: <65/≥65 y; Male/Female (n varies by subgroup)	Appendix Tables 6–9
Sensitivity Analyses (Restricted COX)		Minimize reverse causality and COVID-19 impact	Frailty status/Continuous frailty	RIDs (influenza, OA-LRTI, pneumonia)	Full covariates	n = 406,328	Appendix Table 10
Fine and Gray Competing-Risks Regression		Analyze frailty and mortality in RIDs, accounting for competing death	Frailty status/Continuous frailty	RIDs (influenza, OA-LRTI, pneumonia)	Full covariates	First assessment (n = 423,691)	Appendix Table 11

*Full covariates: age; sex; ethnicity; education level; Townsend deprivation index; smoking status; alcohol consumption status; cumulative dietary risk factor score; PM2.5; NO2; nitrogen oxides; BMI category; sleep duration; sleeplessness; daytime dozing. Abbreviations: RIDs, Respiratory Infectious Diseases; OA-LRTI, Other acute lower respiratory tract infection; PF, physical frailty; FI, frailty index; ΔFI, changes in frailty index; ΔPF, changes in physical frailty.

Appendix Table 3. Baseline characteristics of participants in the first and final assessments based on the frailty index, UK Biobank (UK, 2006–2010 and 2012–2013)

	First assessment				Final assessment			
	Frail	Pre-frail	Non-frail		Frail	Pre-frail	Non-frail	
Characteristic	(n = 25,804)	(n = 144,591)	(n = 253,296)	*P* Value	(n = 630)	(n = 5,794)	(n = 10,424)	*P* Value
Follow-up, (mean (SD)), y	12.45 (3.30)	13.05 (2.66)	13.41 (2.15)	<0.001	8.93 (2.37)	9.48 (1.72)	9.68 (1.36)	<0.001
Age, (mean (SD)), y	58.11 (7.61)	57.26 (8.00)	55.65 (8.11)	<0.001	63.28 (7.25)	62.41 (7.24)	60.52 (7.45)	<0.001
Sex, n (%)				<0.001				0.002
Male	10349 (40.1)	62923 (43.5)	119521 (47.2)		276 (43.8)	2783 (48.0)	5204 (49.9)	
Female	15455 (59.9)	81668 (56.5)	133775 (52.8)		354 (56.2)	3011 (52.0)	5220 (50.1)	
Ethnicity, n (%)				<0.001				0.895
White	23862 (92.5)	135834 (93.9)	240540 (95.0)		613 (97.3)	5652 (97.5)	10173 (97.6)	
Another race	1942 (7.5)	8757 (6.1)	12756 (5.0)		17 (2.7)	142 (2.5)	251 (2.4)	
Education, n (%)				<0.001				<0.001
High	4903 (19.0)	41587 (28.8)	95817 (37.8)		189 (30.0)	2325 (40.1)	4965 (47.6)	
Intermediate	12716 (49.3)	73897 (51.1)	126025 (49.8)		327 (51.9)	2857 (49.3)	4779 (45.8)	
Low	8185 (31.7)	29107 (20.1)	31454 (12.4)		114 (18.1)	612 (10.6)	680 (6.5)	
Smoking status, n (%)				<0.001				<0.001
Never	10993 (42.6)	73551 (50.9)	150513 (59.4)		273 (43.3)	3145 (54.3)	6644 (63.7)	
Previous	10308 (39.9)	54388 (37.6)	80860 (31.9)		300 (47.6)	2251 (38.9)	3199 (30.7)	
Current	4503 (17.5)	16652 (11.5)	21923 (8.7)		57 (9.0)	398 (6.9)	581 (5.6)	
Alcohol status, n (%)				<0.001				<0.001
Never	1855 (7.2)	6594 (4.6)	9262 (3.7)		30 (4.8)	207 (3.6)	333 (3.2)	
Previous	2308 (8.9)	6177 (4.3)	5818 (2.3)		42 (6.7)	216 (3.7)	238 (2.3)	
Current	21641 (83.9)	131820 (91.2)	238216 (94.0)		558 (88.6)	5371 (92.7)	9852 (94.5)	
Townsend deprivation index (mean (SD))	0.10 (3.55)	−1.11 (3.16)	−1.65 (2.89)	<0.001	−0.84 (3.30)	−1.92 (2.76)	−2.19 (2.57)	<0.001
Sleep duration, (mean (SD)), hours/day	6.80 (1.95)	7.04 (1.34)	7.19 (1.03)	<0.001	6.91 (1.82)	7.16 (1.25)	7.25 (0.97)	<0.001

	First assessment				Final assessment			
	Frail	Pre-frail	Non-frail		Frail	Pre-frail	Non-frail	
Characteristic	(n = 25,804)	(n = 144,591)	(n = 253,296)	*P* Value	(n = 630)	(n = 5,794)	(n = 10,424)	*P* Value
Cumulative dietary risk factor score (mean (SD))	5.19 (1.44)	5.02 (1.40)	4.88 (1.39)	<0.001	5.12 (1.33)	4.98 (1.34)	4.82 (1.32)	<0.001
$PM_{2.5}$, μg/m^3, (mean (SD))	10.21 (1.09)	10.03 (1.06)	9.94 (1.04)	<0.001	10.17 (1.09)	10.00 (1.06)	9.94 (1.03)	<0.001
NO_2, μg/m^3, (mean (SD))	28.07 (7.71)	26.93 (7.61)	26.38 (7.59)	<0.001	27.60 (7.14)	26.30 (6.93)	25.87 (6.75)	<0.001
Nitrogen oxides, μg/m^3, (mean (SD))	46.93 (16.44)	44.58 (15.64)	43.39 (15.36)	<0.001	46.07 (15.09)	43.75 (14.67)	42.76 (14.02)	<0.001
BMI category (kg/m^2), n (%)				<0.001				<0.001
Normal weight (18.5 to <25)	4243 (16.4)	39183 (27.1)	95592 (37.7)		112 (18.2)	1688 (29.6)	4326 (41.8)	
Underweight (<18.5)	119 (0.5)	655 (0.5)	1230 (0.5)		3 (0.5)	25 (0.4)	57 (0.6)	
Overweight (25 to <30)	9163 (35.5)	61554 (42.6)	111068 (43.8)		224 (36.5)	2485 (43.5)	4455 (43.0)	
Obese ≥30	12279 (47.6)	43199 (29.9)	45406 (17.9)		275 (44.8)	1513 (26.5)	1523 (14.7)	
RIDs, n (%)				<0.001				<0.001
No	19891 (77.1)	125577 (86.8)	234115 (92.4)		509 (80.8)	5299 (91.5)	9851 (94.5)	
Yes	5913 (22.9)	19014 (13.2)	19181 (7.6)		121 (19.2)	495 (8.5)	573 (5.5)	

Abbreviations: PF, physical frailty; SD, standard deviation;BMI, Body Mass Index; RIDs, Respiratory Infectious Diseases.

Levels of significance: $p < 0.05$ (ANOVA, Kruskal–Wallis H, and chi-squared tests).

Appendix Table 4. Overlap between Physical Frailty and Frailty index

	Physical Frailty			
Frailty index	Frail	Prefrail	Nonfrail	Total
Frail*	4,069 (0.96%)	14,705 (3.47%)	7,030 (1.66%)	2,5804
Pre-frail*	3,991 (0.94%)	61,375 (14.49%)	79,225 (18.70%)	144,591
Non-frail*	1,015 (0.24%)	68,654 (16.20%)	183,627 (43.34%)	253,296
Total*	9,075	144,734	269,882	423,691
Frail†	112 (0.66%)	405 (2.40%)	113 (0.67%)	630
Pre-frail†	233 (1.38%)	3,351 (19.89%)	2,210 (13.12%)	5,794
Non-frail†	64 (0.38%)	4,594 (27.27%)	5,766 (34.22%)	10,424
Total†	409	8,350	8,089	16,848

*The first assessment.

†The final assessment.

Appendix Table 5. Comparison of baseline characteristics between the final assessment characteristics and the first assessment-only characteristics, UK Biobank (UK, 2006–2022)*

Characteristics	First assessment-only (n = 406,843)	Final assessment (n = 16,848)	p value†
Follow-up, (mean (SD)), y	13.79 (2.45)	9.58 (1.55)	<0.001
Age, (mean (SD)), y	56.32 (8.12)	61.27 (7.43)	<0.001
Sex, n (%)			<0.001
Male	184530 (45.4)	8263 (49.0)	
Female	222313 (54.6)	8585 (51.0)	
Ethnicity, n (%)			<0.001
White	383798 (94.3)	16438 (97.6)	
Another race	23045 (5.7)	410 (2.4)	
Education, n (%)			<0.001
High	134828 (33.1)	7479 (44.4)	
Intermediate	204675 (50.3)	7963 (47.3)	
Low	67340 (16.6)	1406 (8.3)	
Smoking status, n (%)			<0.001
Never	224995 (55.3)	10062 (59.7)	
Previous	139806 (34.4)	5750 (34.1)	
Current	42042 (10.3)	1036 (6.1)	
Alcohol status, n (%)			<0.001
Never	17211 (4.2)	500 (3)	
Previous	13905 (3.4)	398 (2.4)	
Current	375727 (92.4)	15950 (94.7)	
Townsend deprivation index (mean (SD))	−1.33 (3.07)	−2.05 (2.68)	<0.001

Characteristics	First assessment-only (n = 406,843)	Final assessment (n = 16,848)	p value†
Sleep duration, (mean (SD)), hours/day	7.11 (1.23)	7.17 (1.09)	<0.001
Cumulative dietary risk factor score (mean (SD))	4.95 (1.4)	4.8 (1.37)	<0.001
$PM_{2.5}$, μg/m^3, (mean (SD))	9.99 (1.05)	9.97 (1.05)	<0.05
NO_2, μg/m^3, (mean (SD))	26.7 (7.64)	26.08 (6.84)	<0.001
Nitrogen oxides, μg/m^3, (mean (SD))	44.04 (15.6)	43.22 (14.31)	<0.001
BMI category (kg/m^2), n (%)			<0.001
Normal weight (18.5 to <25)	132724 (32.6)	6294 (37.4)	
Underweight (<18.5)	1937 (0.5)	67 (0.4)	
Overweight (25 to <30)	174580 (42.9)	7205 (42.8)	
Obese ≥30	97602 (24)	3282 (19.5)	
RIDs, n (%)			<0.001
No	363924 (89.5)	15659 (92.9)	
Yes	42919 (10.5)	1189 (7.1)	

* BMI, Body Mass Index; PF, physical frailty; RIDs, Respiratory Infectious Diseases; SD, standard deviation.

†p<0.05 statistically significant by ANOVA, Kruskal–Wallis H, and chi-squared tests.

Appendix Table 6. The hazard ratios of incident RIDs associated with the first and final assessment of frailty in the ≥65 y subgroup, UK Biobank (UK, 2006–2022)

	First assessment					Final assessment				
		Model 1		Model 2			Model 1		Model 2	
RIDs	Events/all participants	HR (95% CI)	*P* -value	HR (95% CI)	*P* -value	Events/all participants	HR (95% CI)	*P* -value	HR (95% CI)	*P* -value
RIDs										
Non-frail*	16399/224303	1.00 (reference)	-	1.00 (reference)	-	238/5575	1.00 (reference)	-	1.00 (reference)	-
Pre-frail*	12686/114528	1.53 (1.49, 1.56)	<0.001	1.32 (1.29, 1.35)	<0.001	273/4965	1.26 (1.06, 1.50)	0.009	1.10 (0.92, 1.32)	0.284
Frail*	1512/6987	3.09 (2.93, 3.26)	<0.001	2.02 (1.91, 2.13)	<0.001	36/236	3.77 (2.65, 5.35)	<0.001	2.38 (1.63, 3.47)	<0.001
Non-frail†	13838/213135	1.00 (reference)	-	1.00 (reference)	-	285/7122	1.00 (reference)	-	1.00 (reference)	-
Pre-frail†	12727/113028	1.72 (1.68, 1.77)	<0.001	1.53 (1.49, 1.56)	<0.001	207/3331	1.53 (1.28, 1.83)	<0.001	1.33 (1.11, 1.60)	0.003
Frail†	4032/19655	3.21 (3.10, 3.32)	<0.001	2.36 (2.27, 2.45)	<0.001	55/323	4.49 (3.36, 5.99)	<0.001	3.18 (2.33, 4.34)	<0.001
per 1-point increase for PF		1.43 (1.41, 1.44)	<0.001	1.26 (1.24, 1.27)	<0.001		1.40 (1.27, 1.54)	<0.001	1.24 (1.12, 1.37)	<0.001
per 0.1-point increase for FI		1.67 (1.65, 1.70)	<0.001	1.48 (1.46, 1.50)	<0.001		1.87 (1.67, 2.10)	<0.001	1.60 (1.42, 1.81)	<0.001
Subgroups of RIDs										
Influenza										
Non-frail*	945/224303	1.00 (reference)	-	1.00 (reference)	-	3/5575	1.00 (reference)	-	1.00 (reference)	-
Pre-frail*	697/114528	1.44 (1.30, 1.59)	<0.001	1.32 (1.19, 1.46)	<0.001	11/4965	4.13 (1.15, 14.85)	0.030	3.64 (1.00, 13.23)	0.050
Frail*	94/6987	3.17 (2.56, 3.92)	<0.001	2.41 (1.93, 3.02)	<0.001	2/236	15.07 (2.51, 90.65)	0.003	9.67 (1.33, 70.07)	0.025
Non-frail†	858/213135	1.00 (reference)	-	1.00 (reference)	-	8/7122	1.00 (reference)	-	1.00 (reference)	-
Pre-frail†	661/113028	1.45 (1.31, 1.60)	<0.001	1.35 (1.21, 1.49)	<0.001	5/3331	1.33 (0.44, 4.09)	0.615	1.06 (0.34, 3.35)	0.921
Frail†	217/19655	2.73 (2.35, 3.17)	<0.001	2.24 (1.91, 2.63)	<0.001	3/323	8.03 (2.12, 30.44)	0.002	4.76 (1.07, 21.25)	0.041
per 1-point increase for PF		1.38 (1.31, 1.46)	<0.001	1.28 (1.21, 1.36)	<0.001		1.94 (1.22, 3.09)	0.005	1.72 (1.05, 2.81)	0.030

	First assessment					Final assessment				
		Model 1		Model 2			Model 1		Model 2	
RIDs	Events/all participants	HR (95% CI)	*P* -value	HR (95% CI)	*P* - value	Events/all participants	HR (95% CI)	*P* - value	HR (95% CI)	*P* - value
per 0.1-point increase for FI		1.51 (1.43, 1.60)	<0.001	1.40 (1.32, 1.49)	<0.001		2.87 (1.62, 5.10)	<0.001	2.45 (1.29, 4.64)	0.006
OA-LRTI										
Non-frail*	9681/224303	1.00 (reference)	-	1.00 (reference)	-	142/5575	1.00 (reference)	-	1.00 (reference)	-
Pre-frail*	7248/114528	1.46 (1.41, 1.50)	<0.001	1.27 (1.23, 1.31)	<0.001	122/4965	0.93 (0.73, 1.18)	0.547	0.85 (0.66, 1.09)	0.190
Frail*	789/6987	2.59 (2.41, 2.79)	<0.001	1.76 (1.63, 1.89)	<0.001	15/236	2.43 (1.42, 4.14)	0.001	1.82 (1.02, 3.24)	0.042
Non-frail†	7968/213135	1.00 (reference)	-	1.00 (reference)	-	146/7122	1.00 (reference)	-	1.00 (reference)	-
Pre-frail†	7491/113028	1.74 (1.69, 1.80)	<0.001	1.56 (1.51, 1.62)	<0.001	107/3331	1.52 (1.19, 1.95)	<0.001	1.42 (1.09, 1.83)	0.008
Frail†	2259/19655	3.02 (2.88, 3.17)	<0.001	2.31 (2.19, 2.42)	<0.001	26/323	3.85 (2.53, 5.85)	<0.001	3.25 (2.08, 5.08)	<0.001
per 1-point increase for PF		1.36 (1.33, 1.38)	<0.001	1.21 (1.19, 1.23)	<0.001		1.14 (0.99, 1.32)	0.073	1.07 (0.92, 1.24)	0.395
per 0.1-point increase for FI		1.64 (1.61, 1.67)	<0.001	1.47 (1.45, 1.50)	<0.001		1.74 (1.48, 2.04)	<0.001	1.66 (1.39, 1.97)	<0.001
Pneumonia										
Non-frail*	7216/224303	1.00 (reference)	-	1.00 (reference)	-	108/5575	1.00 (reference)	-	1.00 (reference)	-
Pre-frail*	6299/114528	1.72 (1.67, 1.78)	<0.001	1.45 (1.40, 1.50)	<0.001	160/4965	1.63 (1.28, 2.09)	<0.001	1.38 (1.07, 1.77)	0.012
Frail*	911/6987	4.21 (3.93, 4.51)	<0.001	2.51 (2.34, 2.71)	<0.001	24/236	5.62 (3.61, 8.76)	<0.001	2.92 (1.80, 4.74)	<0.001
Non-frail†	6183/213135	1.00 (reference)	-	1.00 (reference)	-	144/7122	1.00 (reference)	-	1.00 (reference)	-
Pre-frail†	6026/113028	1.79 (1.73, 1.86)	<0.001	1.54 (1.48, 1.59)	<0.001	113/3331	1.64 (1.28, 2.10)	<0.001	1.34 (1.04, 1.74)	0.025
Frail†	2217/19655	3.79 (3.61, 3.98)	<0.001	2.57 (2.44, 2.71)	<0.001	35/323	5.60 (3.86, 8.11)	<0.001	3.57 (2.39, 5.33)	<0.001
per 1-point increase for PF		1.58 (1.55, 1.60)	<0.001	1.36 (1.33, 1.38)	<0.001		1.67 (1.48, 1.88)	<0.001	1.38 (1.22, 1.57)	<0.001
per 0.1-point increase for FI		1.79 (1.76, 1.83)	<0.001	1.53 (1.50, 1.57)	<0.001		2.04 (1.75, 2.38)	<0.001	1.61 (1.36, 1.90)	<0.001

	First assessment					Final assessment				
		Model 1		Model 2			Model 1		Model 2	
RIDs	Events/all participants	HR (95% CI)	*P* -value	HR (95% CI)	*P* -value	Events/all participants	HR (95% CI)	*P* -value	HR (95% CI)	*P* -value

Model 1: Adjusted for age, sex. Model 2: Adjusted for all covariates. FI, frailty index; OA-LRTI, Other acute lower respiratory tract infection; PF, physical frailty; RIDs, Respiratory Infectious Diseases.

HRs and 95% CIs were estimated using Cox proportional hazards models; corresponding *P* values are shown in the table. A two-sided $p < 0.05$ was considered statistically significant.

*Grouped according to physical frailty.

†Grouped according to frailty index.

Appendix Table 7. The hazard ratios of incident RIDs associated with the first and final assessment of frailty in the <65 y subgroup, UK Biobank (UK, 2006–2022)

	First assessment					Final assessment				
		Model 1		Model 2			Model 1		Model 2	
RIDs	Events/all participants	HR (95% CI)	*P* -value	HR (95% CI)	*P* -value	Events/all participants	HR (95% CI)	*P* -value	HR (95% CI)	*P* -value
RIDs										
Non-frail[a]	6682/45579	1.00 (reference)	-	1.00 (reference)	-	224/2514	1.00 (reference)	-	1.00 (reference)	-
Pre-frail[a]	6148/30206	1.47 (1.42, 1.52)	<0.001	1.32 (1.27, 1.37)	<0.001	382/3385	1.29 (1.10, 1.53)	0.002	1.22 (1.03, 1.45)	0.021
Frail[a]	681/2088	2.64 (2.44, 2.85)	<0.001	1.99 (1.83, 2.16)	<0.001	36/173	2.51 (1.76, 3.57)	<0.001	2.12 (1.47, 3.06)	<0.001
Non-frail[b]	5343/40161	1.00 (reference)	-	1.00 (reference)	-	288/3302	1.00 (reference)	-	1.00 (reference)	-
Pre-frail[b]	6287/31563	1.56 (1.51, 1.62)	<0.001	1.44 (1.39, 1.49)	<0.001	288/2463	1.34 (1.14, 1.58)	<0.001	1.23 (1.04, 1.45)	0.018
Frail[b]	1881/6149	2.64 (2.51, 2.79)	<0.001	2.16 (2.04, 2.28)	<0.001	66/307	2.62 (2.00, 3.42)	<0.001	2.21 (1.66, 2.94)	<0.001
per 1-point increase for PF		1.37 (1.34, 1.39)	<0.001	1.25 (1.23, 1.28)	<0.001		1.33 (1.21, 1.45)	<0.001	1.23 (1.12, 1.36)	<0.001
per 0.1-point increase for FI		1.56 (1.53, 1.60)	<0.001	1.43 (1.40, 1.47)	<0.001		1.48 (1.33, 1.65)	<0.001	1.34 (1.20, 1.51)	<0.001

	First assessment					Final assessment				
		Model 1		Model 2			Model 1		Model 2	
RIDs	Events/all participants	HR (95% CI)	*P* -value	HR (95% CI)	*P* - value	Events/all participants	HR (95% CI)	*P* - value	HR (95% CI)	*P* - value
Subgroups of RIDs										
Influenza										
Non-frail[a]	264/45579	1.00 (reference)	-	1.00 (reference)	-	6/2514	1.00 (reference)	-	1.00 (reference)	-
Pre-frail[a]	234/30206	1.34 (1.12, 1.59)	0.001	1.24 (1.04, 1.49)	0.019	9/3385	1.05 (0.37, 2.96)	0.928	0.94 (0.32, 2.70)	0.905
Frail[a]	38/2088	3.15 (2.24, 4.44)	<0.001	2.51 (1.76, 3.60)	<0.001	0/173	0.00 (0.00, Inf)	0.997	0.00 (0.00, Inf)	0.998
Non-frail[b]	199/40161	1.00 (reference)	-	1.00 (reference)	-	10/3302	1.00 (reference)	-	1.00 (reference)	-
Pre-frail[b]	263/31563	1.67 (1.39, 2.01)	<0.001	1.59 (1.32, 1.92)	<0.001	4/2463	0.51 (0.16, 1.64)	0.261	0.43 (0.13, 1.41)	0.164
Frail[b]	74/6149	2.42 (1.85, 3.17)	<0.001	2.10 (1.59, 2.79)	<0.001	1/307	0.99 (0.13, 7.81)	0.996	0.70 (0.08, 6.13)	0.749
per 1-point increase for PF		1.42 (1.30, 1.55)	<0.001	1.34 (1.21, 1.47)	<0.001		0.79 (0.40, 1.58)	0.510	0.73 (0.36, 1.47)	0.381
per 0.1-point increase for FI		1.52 (1.38, 1.69)	<0.001	1.44 (1.29, 1.61)	<0.001		0.94 (0.43, 2.05)	0.874	0.82 (0.36, 1.87)	0.636
OA-LRTI										
Non-frail[a]	3389/45579	1.00 (reference)	-	1.00 (reference)	-	100/2514	1.00 (reference)	-	1.00 (reference)	-
Pre-frail[a]	3060/30206	1.40 (1.33, 1.47)	<0.001	1.26 (1.20, 1.33)	<0.001	179/3385	1.36 (1.06, 1.74)	0.014	1.23 (0.96, 1.59)	0.101
Frail[a]	342/2088	2.37 (2.12, 2.65)	<0.001	1.83 (1.63, 2.06)	<0.001	13/173	1.95 (1.10, 3.49)	0.023	1.57 (0.87, 2.85)	0.136
Non-frail[b]	2627/40161	1.00 (reference)	-	1.00 (reference)	-	132/3302	1.00 (reference)	-	1.00 (reference)	-
Pre-frail[b]	3186/31563	1.58 (1.50, 1.66)	<0.001	1.45 (1.38, 1.53)	<0.001	126/2463	1.29 (1.01, 1.64)	0.044	1.17 (0.91, 1.51)	0.216
Frail[b]	978/6149	2.60 (2.41, 2.79)	<0.001	2.15 (1.99, 2.32)	<0.001	34/307	2.88 (1.98, 4.21)	<0.001	2.41 (1.62, 3.59)	<0.001
per 1-point increase for PF		1.31 (1.28, 1.35)	<0.001	1.21 (1.18, 1.25)	<0.001		1.30 (1.14, 1.48)	<0.001	1.20 (1.05, 1.39)	0.009
per 0.1-point increase for FI		1.55 (1.51, 1.60)	<0.001	1.44 (1.39, 1.48)	<0.001		1.53 (1.30, 1.80)	<0.001	1.38 (1.17, 1.64)	<0.001
Pneumonia										

	First assessment					Final assessment				
		Model 1		Model 2			Model 1		Model 2	
RIDs	Events/all participants	HR (95% CI)	*P* -value	HR (95% CI)	*P* -value	Events/all participants	HR (95% CI)	*P* -value	HR (95% CI)	*P* -value
Non-frail[a]	4005/45579	1.00 (reference)	-	1.00 (reference)	-	139/2514	1.00 (reference)	-	1.00 (reference)	-
Pre-frail[a]	3905/30206	1.56 (1.49, 1.63)	<0.001	1.39 (1.33, 1.45)	<0.001	248/3385	1.35 (1.10, 1.67)	0.005	1.31 (1.05, 1.62)	0.015
Frail[a]	459/2088	2.95 (2.68, 3.25)	<0.001	2.16 (1.95, 2.39)	<0.001	29/173	3.22 (2.16, 4.81)	<0.001	2.82 (1.86, 4.29)	<0.001
Non-frail[b]	3230/40161	1.00 (reference)	-	1.00 (reference)	-	176/3302	1.00 (reference)	-	1.00 (reference)	-
Pre-frail[b]	3914/31563	1.60 (1.52, 1.67)	<0.001	1.46 (1.39, 1.53)	<0.001	199/2463	1.50 (1.22, 1.84)	<0.001	1.38 (1.12, 1.70)	0.003
Frail[b]	1225/6149	2.79 (2.61, 2.98)	<0.001	2.24 (2.09, 2.40)	<0.001	41/307	2.58 (1.84, 3.63)	<0.001	2.20 (1.53, 3.17)	<0.001
per 1-point increase for PF		1.43 (1.40, 1.46)	<0.001	1.30 (1.27, 1.33)	<0.001		1.42 (1.27, 1.58)	<0.001	1.32 (1.18, 1.48)	<0.001
per 0.1-point increase for FI		1.60 (1.56, 1.65)	<0.001	1.46 (1.42, 1.50)	<0.001		1.53 (1.33, 1.75)	<0.001	1.38 (1.19, 1.59)	<0.001

Abbreviations: RIDs, Respiratory Infectious Diseases; OA-LRTI, Other acute lower respiratory tract infection; PF, physical frailty; FI, frailty index.

HRs and 95% CIs were estimated using Cox proportional hazards models; corresponding *P* values are shown in the table. A two-sided $p < 0.05$ was considered statistically significant.

[a]: Grouped according to physical frailty.

[b]: Grouped according to frailty index.

Model 1: Adjusted for age, sex.

Model 2: Adjusted for all covariates.

Appendix Table 8. The hazard ratios of incident RIDs associated with the first and final assessment of frailty in men, UK Biobank (UK, 2006–2022)

	First assessment					Final assessment				
		Model 1		Model 2			Model 1		Model 2	
RIDs	Events/all participants	HR (95% CI)	*P* -value	HR (95% CI)	*P* -value	Events/all participants	HR (95% CI)	*P* -value	HR (95% CI)	*P* -value
RIDs										
Non-frail[a]	12501/127389	1.00 (reference)	-	1.00 (reference)	-	263/3945	1.00 (reference)	-	1.00 (reference)	-
Pre-frail[a]	9081/62077	1.48 (1.44, 1.52)	<0.001	1.30 (1.27, 1.34)	<0.001	384/4400	1.41 (1.20, 1.65)	<0.001	1.30 (1.11, 1.53)	0.001
Frail[a]	905/3327	2.84 (2.66, 3.04)	<0.001	1.94 (1.81, 2.08)	<0.001	34/240	2.96 (2.07, 4.23)	<0.001	2.15 (1.48, 3.11)	<0.001
Non-frail[b]	10440/119521	1.00 (reference)	-	1.00 (reference)	-	357/5220	1.00 (reference)	-	1.00 (reference)	-
Pre-frail[b]	9485/62923	1.64 (1.60, 1.69)	<0.001	1.46 (1.42, 1.51)	<0.001	269/3011	1.25 (1.07, 1.47)	0.006	1.09 (0.93, 1.29)	0.290
Frail[b]	2562/10349	2.79 (2.67, 2.92)	<0.001	2.10 (2.01, 2.20)	<0.001	55/354	2.61 (1.96, 3.47)	<0.001	1.86 (1.37, 2.52)	<0.001
per 1-point increase for PF		1.41 (1.39, 1.42)	<0.001	1.26 (1.24, 1.27)	<0.001		1.36 (1.27, 1.46)	<0.001	1.25 (1.17, 1.35)	<0.001
per 0.1-point increase for FI		1.64 (1.62, 1.66)	<0.001	1.47 (1.45, 1.49)	<0.001		1.66 (1.53, 1.80)	<0.001	1.48 (1.36, 1.62)	<0.001
Subgroups of RIDs										
Influenza										
Non-frail[a]	544/127389	1.00 (reference)	-	1.00 (reference)	-	6/3945	1.00 (reference)	-	1.00 (reference)	-
Pre-frail[a]	389/62077	1.45 (1.28, 1.66)	<0.001	1.36 (1.19, 1.56)	<0.001	5/4400	0.88 (0.27, 2.90)	0.833	0.84 (0.25, 2.79)	0.771
Frail[a]	48/3327	3.31 (2.46, 4.44)	<0.001	2.67 (1.96, 3.64)	<0.001	1/240	4.14 (0.50, 34.70)	0.190	5.14 (0.53, 49.94)	0.158
Non-frail[b]	486/119521	1.00 (reference)	-	1.00 (reference)	-	9/5220	1.00 (reference)	-	1.00 (reference)	-
Pre-frail[b]	400/62923	1.53 (1.34, 1.75)	<0.001	1.45 (1.27, 1.66)	<0.001	3/3011	0.62 (0.17, 2.32)	0.477	0.60 (0.16, 2.29)	0.453
Frail[b]	95/10349	2.19 (1.76, 2.73)	<0.001	1.88 (1.49, 2.37)	<0.001	0/354	0.00 (0.00, Inf)	0.997	0.00 (0.00, Inf)	0.998
per 1-point increase for PF		1.39 (1.33, 1.46)	<0.001	1.29 (1.23, 1.36)	<0.001		1.38 (0.96, 1.98)	0.079	1.20 (0.79, 1.80)	0.382

	First assessment					Final assessment				
		Model 1		Model 2			Model 1		Model 2	
RIDs	Events/all participants	HR (95% CI)	*P* -value	HR (95% CI)	*P* -value	Events/all participants	HR (95% CI)	*P* -value	HR (95% CI)	*P* -value
per 0.1-point increase for FI		1.52 (1.44, 1.59)	<0.001	1.41 (1.34, 1.49)	<0.001		1.82 (1.15, 2.90)	<0.05	1.53 (0.94, 2.50)	0.087
OA-LRTI										
Non-frail[a]	6579/127389	1.00 (reference)	-	1.00 (reference)	-	122/3945	1.00 (reference)	-	1.00 (reference)	-
Pre-frail[a]	4555/62077	1.39 (1.34, 1.44)	<0.001	1.24 (1.19, 1.29)	<0.001	162/4400	1.28 (1.01, 1.62)	0.043	1.17 (0.92, 1.49)	0.190
Frail[a]	432/3327	2.44 (2.21, 2.68)	<0.001	1.74 (1.57, 1.92)	<0.001	12/240	2.12 (1.17, 3.84)	0.013	1.63 (0.88, 3.00)	0.117
Non-frail[b]	5351/119521	1.00 (reference)	-	1.00 (reference)	-	158/5220	1.00 (reference)	-	1.00 (reference)	-
Pre-frail[b]	4921/62923	1.66 (1.59, 1.72)	<0.001	1.49 (1.44, 1.55)	<0.001	112/3011	1.18 (0.92, 1.50)	0.191	1.04 (0.80, 1.33)	0.785
Frail[b]	1294/10349	2.66 (2.51, 2.83)	<0.001	2.07 (1.94, 2.21)	<0.001	26/354	2.70 (1.78, 4.10)	<0.001	2.01 (1.29, 3.14)	0.002
per 1-point increase for PF		1.34 (1.33, 1.36)	<0.001	1.21 (1.19, 1.23)	<0.001		1.22 (1.10, 1.36)	<0.001	1.14 (1.02, 1.27)	0.024
per 0.1-point increase for FI		1.62 (1.59, 1.64)	<0.001	1.47 (1.44, 1.49)	<0.001		1.63 (1.45, 1.83)	<0.001	1.51 (1.32, 1.71)	<0.001
Pneumonia										
Non-frail[a]	6845/127389	1.00 (reference)	-	1.00 (reference)	-	156/3945	1.00 (reference)	-	1.00 (reference)	-
Pre-frail[a]	5538/62077	1.61 (1.56, 1.67)	<0.001	1.40 (1.35, 1.45)	<0.001	261/4400	1.58 (1.30, 1.93)	<0.001	1.46 (1.19, 1.79)	<0.001
Frail[a]	626/3327	3.45 (3.18, 3.75)	<0.001	2.22 (2.04, 2.42)	<0.001	25/240	3.53 (2.31, 5.39)	<0.001	2.51 (1.62, 3.90)	<0.001
Non-frail[b]	5772/119521	1.00 (reference)	-	1.00 (reference)	-	217/5220	1.00 (reference)	-	1.00 (reference)	-
Pre-frail[b]	5584/62923	1.68 (1.62, 1.74)	<0.001	1.47 (1.42, 1.53)	<0.001	187/3011	1.39 (1.14, 1.70)	0.001	1.22 (0.99, 1.50)	0.058
Frail[b]	1653/10349	3.06 (2.90, 3.24)	<0.001	2.20 (2.08, 2.33)	<0.001	38/354	2.81 (1.98, 3.97)	<0.001	2.04 (1.40, 2.95)	<0.001
per 1-point increase for PF		1.52 (1.50, 1.54)	<0.001	1.34 (1.32, 1.36)	<0.001		1.53 (1.40, 1.67)	<0.001	1.39 (1.27, 1.52)	<0.001
per 0.1-point increase for FI		1.73 (1.70, 1.76)	<0.001	1.51 (1.49, 1.54)	<0.001		1.73 (1.56, 1.92)	<0.001	1.52 (1.35, 1.70)	<0.001

	First assessment					Final assessment				
		Model 1		Model 2			Model 1		Model 2	
RIDs	Events/all participants	HR (95% CI)	*P* -value	HR (95% CI)	*P* -value	Events/all participants	HR (95% CI)	*P* -value	HR (95% CI)	*P* -value

Abbreviations: RIDs, Respiratory Infectious Diseases; OA-LRTI, Other acute lower respiratory tract infection; PF, physical frailty; FI, frailty index.

HRs and 95% CIs were estimated using Cox proportional hazards models; corresponding *P* values are shown in the table. A two-sided $p < 0.05$ was considered statistically significant.

[a]: Grouped according to physical frailty.

[b]: Grouped according to frailty index.

Model 1: Adjusted for age.

Model 2: Adjusted for all covariates.

Appendix Table 9. The hazard ratios of incident RIDs associated with the first and final assessment of frailty in women, UK Biobank (UK, 2006–2022)

	First assessment					Final assessment				
		Model 1		Model 2			Model 1		Model 2	
RIDs	Events/all participants	HR (95% CI)	*P* -value	HR (95% CI)	*P* -value	Events/all participants	HR (95% CI)	*P* -value	HR (95% CI)	*P* -value
RIDs										
Non-frail[a]	10580/142493	1.00 (reference)	-	1.00 (reference)	-	199/3945	1.00 (reference)	-	1.00 (reference)	-
Pre-frail[a]	9753/82657	1.40 (1.25, 1.56)	<0.001	1.25 (1.12, 1.41)	<0.001	271/4400	1.28 (1.14, 1.45)	<0.001	1.17 (1.04, 1.33)	0.011
Frail[a]	1288/5748	3.10 (2.47, 3.89)	<0.001	2.28 (1.79, 2.90)	<0.001	38/240	3.04 (2.37, 3.90)	<0.001	2.27 (1.75, 2.95)	<0.001
Non-frail[b]	8741/81668	1.00 (reference)	-	1.00 (reference)	-	216/5220	1.00 (reference)	-	1.00 (reference)	-
Pre-frail[b]	9529/81668	1.68 (1.65, 1.71)	<0.001	1.50 (1.47, 1.53)	<0.001	226/3011	1.44 (1.27, 1.62)	<0.001	1.28 (1.13, 1.45)	<0.001
Frail[b]	3351/15455	3.02 (2.93, 3.11)	<0.001	2.29 (2.22, 2.37)	<0.001	66/354	3.31 (2.72, 4.03)	<0.001	2.59 (2.10, 3.19)	<0.001
per 1-point increase for PF		1.41 (1.39, 1.42)	<0.001	1.26 (1.24, 1.27)	<0.001		1.36 (1.27, 1.46)	<0.001	1.25 (1.17, 1.35)	<0.001

	First assessment					Final assessment				
		Model 1		Model 2			Model 1		Model 2	
RIDs	Events/all participants	HR (95% CI)	*P* -value	HR (95% CI)	*P* -value	Events/all participants	HR (95% CI)	*P* -value	HR (95% CI)	*P* -value
per 0.1-point increase for FI		1.64 (1.62, 1.66)	<0.001	1.47 (1.45, 1.49)	<0.001		1.66 (1.53, 1.80)	<0.001	1.48 (1.36, 1.62)	<0.001
Subgroups of RIDs										
Influenza										
Non-frail[a]	665/142493	1.00 (reference)	-	1.00 (reference)	-	3/3945	1.00 (reference)	-	1.00 (reference)	-
Pre-frail[a]	542/82657	1.49 (1.44, 1.54)	<0.001	1.30 (1.25, 1.35)	<0.001	15/4400	2.04 (0.92, 4.50)	0.078	1.81 (0.81, 4.05)	0.146
Frail[a]	84/5748	2.60 (2.40, 2.81)	<0.001	1.81 (1.67, 1.97)	<0.001	1/240	4.04 (0.87, 18.81)	0.075	2.36 (0.45, 12.47)	0.312
Non-frail[b]	571/81668	1.00 (reference)	-	1.00 (reference)	-	9/5220	1.00 (reference)	-	1.00 (reference)	-
Pre-frail[b]	524/81668	1.51 (1.38, 1.65)	<0.001	1.41 (1.29, 1.54)	<0.001	6/3011	0.85 (0.38, 1.91)	0.696	0.73 (0.32, 1.65)	0.443
Frail[b]	196/15455	2.64 (2.31, 3.00)	<0.001	2.19 (1.90, 2.51)	<0.001	4/354	3.35 (1.12, 9.98)	<0.05	2.09 (0.63, 6.95)	0.228
per 1-point increase for PF		1.39 (1.33, 1.46)	<0.001	1.29 (1.23, 1.36)	<0.001		1.38 (0.96, 1.98)	0.079	1.20 (0.79, 1.80)	0.382
per 0.1-point increase for FI		1.52 (1.44, 1.59)	<0.001	1.41 (1.34, 1.49)	<0.001		1.82 (1.15, 2.90)	<0.05	1.53 (0.94, 2.50)	0.087
OA-LRTI										
Non-frail[a]	6491/142493	1.00 (reference)	-	1.00 (reference)	-	120/3945	1.00 (reference)	-	1.00 (reference)	-
Pre-frail[a]	5753/82657	1.74 (1.67, 1.81)	<0.001	1.48 (1.42, 1.55)	<0.001	139/4400	1.12 (0.94, 1.33)	0.194	1.03 (0.86, 1.22)	0.760
Frail[a]	699/5748	3.99 (3.70, 4.32)	<0.001	2.56 (2.36, 2.79)	<0.001	16/240	2.13 (1.44, 3.16)	<0.001	1.64 (1.09, 2.48)	<0.05
Non-frail[b]	5244/81668	1.00 (reference)	-	1.00 (reference)	-	120/5220	1.00 (reference)	-	1.00 (reference)	-
Pre-frail[b]	5756/81668	1.70 (1.66, 1.75)	<0.001	1.54 (1.49, 1.58)	<0.001	121/3011	1.40 (1.18, 1.67)	<0.001	1.28 (1.07, 1.53)	0.007
Frail[b]	1943/15455	2.89 (2.78, 3.01)	<0.001	2.26 (2.17, 2.36)	<0.001	34/354	3.29 (2.49, 4.36)	<0.001	2.77 (2.06, 3.73)	<0.001
per 1-point increase for PF		1.34 (1.33, 1.36)	<0.001	1.21 (1.19, 1.23)	<0.001		1.22 (1.10, 1.36)	<0.001	1.14 (1.02, 1.27)	0.024

	First assessment					Final assessment				
		Model 1		Model 2			Model 1		Model 2	
RIDs	Events/all participants	HR (95% CI)	*P* -value	HR (95% CI)	*P* -value	Events/all participants	HR (95% CI)	*P* -value	HR (95% CI)	*P* -value
per 0.1-point increase for FI		1.62 (1.59, 1.64)	<0.001	1.47 (1.44, 1.49)	<0.001		1.63 (1.45, 1.83)	<0.001	1.51 (1.32, 1.71)	<0.001
Pneumonia										
Non-frail[a]	4376/142493	1.00 (reference)	-	1.00 (reference)	-	91/3945	1.00 (reference)	-	1.00 (reference)	-
Pre-frail[a]	4666/82657	1.55 (1.51, 1.60)	<0.001	1.35 (1.31, 1.39)	<0.001	147/4400	1.47 (1.26, 1.73)	<0.001	1.34 (1.14, 1.58)	<0.001
Frail[a]	744/5748	3.03 (2.86, 3.21)	<0.001	2.06 (1.94, 2.19)	<0.001	28/240	4.12 (3.06, 5.55)	<0.001	2.96 (2.16, 4.05)	<0.001
Non-frail[b]	3641/81668	1.00 (reference)	-	1.00 (reference)	-	103/5220	1.00 (reference)	-	1.00 (reference)	-
Pre-frail[b]	4356/81668	1.73 (1.68, 1.78)	<0.001	1.52 (1.47, 1.56)	<0.001	125/3011	1.58 (1.35, 1.84)	<0.001	1.38 (1.17, 1.62)	<0.001
Frail[b]	1789/15455	3.39 (3.26, 3.53)	<0.001	2.45 (2.35, 2.56)	<0.001	38/354	3.53 (2.75, 4.55)	<0.001	2.67 (2.04, 3.50)	<0.001
per 1-point increase for PF		1.52 (1.50, 1.54)	<0.001	1.34 (1.32, 1.36)	<0.001		1.53 (1.40, 1.67)	<0.001	1.39 (1.27, 1.52)	<0.001
per 0.1-point increase for FI		1.73 (1.70, 1.53)	<0.001	1.51 (1.49, 1.54)	<0.001		1.73 (1.56, 1.92)	<0.001	1.52 (1.35, 1.70)	<0.001

Abbreviations: RIDs, Respiratory Infectious Diseases; OA-LRTI, Other acute lower respiratory tract infection; PF, physical frailty; FI, frailty index.

HRs and 95% CIs were estimated using Cox proportional hazards models; corresponding *P* values are shown in the table. A two-sided $p < 0.05$ was considered statistically significant.

[a]: Grouped according to physical frailty.

[b]: Grouped according to frailty index.

Model 1: Adjusted for age.

Model 2: Adjusted for all covariates.

Appendix Table 10. Sensitivity analysis with exclusion of early outcomes (≤2 y) and post-2020 cases, UK Biobank (UK, 2006–2020)

	Events/all	Model 1		Model 2	
RIDs	participants	HR (95% CI)	*P* -value	HR (95% CI)	*P* -value
RIDs					
Non-frail[a]	11381/260803	1.00 (reference)	-	1.00 (reference)	-
Pre-frail[a]	14002/137281	1.53 (1.49, 1.56)	<0.001	1.35 (1.31, 1.39)	<0.001
Frail[a]	1362/8244	3.10 (2.93, 3.28)	<0.001	2.18 (2.05, 2.32)	<0.001
Non-frail[b]	11579/245694	1.00 (reference)	-	1.00 (reference)	-
Pre-frail[b]	11595/137172	1.71 (1.67, 1.76)	<0.001	1.54 (1.50, 1.58)	<0.001
Frail[b]	3571/23462	3.10 (2.98, 3.22)	<0.001	2.37 (2.27, 2.47)	<0.001
per 1-point increase for PF		1.42 (1.41, 1.44)	<0.001	1.28 (1.26, 1.30)	<0.001
per 0.1-point increase for FI		1.67 (1.64, 1.69)	<0.001	1.50 (1.47, 1.52)	<0.001
Subgroups of RIDs					
Influenza					
Non-frail[a]	661/260803	1.00 (reference)	-	1.00 (reference)	-
Pre-frail[a]	838/137281	1.48 (1.34, 1.64)	<0.001	1.33 (1.19, 1.49)	<0.001
Frail[a]	94/8244	3.31 (2.67, 4.11)	<0.001	2.59 (2.04, 3.29)	<0.001
Non-frail[b]	711/245694	1.00 (reference)	-	1.00 (reference)	-
Pre-frail[b]	670/137172	1.62 (1.46, 1.80)	<0.001	1.46 (1.30, 1.64)	<0.001
Frail[b]	212/23462	2.88 (2.46, 3.36)	<0.001	2.46 (2.06, 2.92)	<0.001
per 1-point increase for PF		1.43 (1.36, 1.51)	<0.001	1.33 (1.25, 1.41)	<0.001
per 0.1-point increase for FI		1.58 (1.49, 1.67)	<0.001	1.48 (1.38, 1.58)	<0.001
OA-LRTI					
Non-frail[a]	6567/260803	1.00 (reference)	-	1.00 (reference)	-
Pre-frail[a]	8357/137281	1.68 (1.63, 1.75)	<0.001	1.47 (1.41, 1.52)	<0.001
Frail[a]	746/8244	3.90 (3.62, 4.19)	<0.001	2.59 (2.39, 2.81)	<0.001
Non-frail[b]	6801/245694	1.00 (reference)	-	1.00 (reference)	-
Pre-frail[b]	6824/137172	1.77 (1.71, 1.84)	<0.001	1.56 (1.50, 1.62)	<0.001
Frail[b]	2045/23462	3.50 (3.33, 3.69)	<0.001	2.58 (2.44, 2.73)	<0.001
per 1-point increase for PF		1.37 (1.34, 1.39)	<0.001	1.24 (1.21, 1.26)	<0.001
per 0.1-point increase for FI		1.63 (1.60, 1.66)	<0.001	1.48 (1.45, 1.51)	<0.001
Pneumonia					
Non-frail[a]	5917/260803	1.00 (reference)	-	1.00 (reference)	-
Pre-frail[a]	6440/137281	1.45 (1.41, 1.50)	<0.001	1.29 (1.24, 1.34)	<0.001
Frail[a]	830/8244	2.73 (2.53, 2.94)	<0.001	1.96 (1.80, 2.13)	<0.001
Non-frail[b]	5331/245694	1.00 (reference)	-	1.00 (reference)	-
Pre-frail[b]	5810/137172	1.71 (1.66, 1.77)	<0.001	1.56 (1.50, 1.62)	<0.001
Frail[b]	2046/23462	2.94 (2.80, 3.09)	<0.001	2.30 (2.17, 2.43)	<0.001

RIDs	Events/all participants	Model 1		Model 2	
		HR (95% CI)	*P* -value	HR (95% CI)	*P* -value
per 1-point increase for PF		1.54 (1.51, 1.57)	<0.001	1.37 (1.34, 1.39)	<0.001
per 0.1-point increase for FI		1.76 (1.72, 1.79)	<0.001	1.55 (1.52, 1.59)	<0.001

Abbreviations: RIDs, Respiratory Infectious Diseases; OA-LRTI, Other acute lower respiratory tract infection; PF, physical frailty; FI, frailty index.

HRs and 95% CIs were estimated using Cox proportional hazards models; corresponding *P* values are shown in the table. A two-sided $p < 0.05$ was considered statistically significant.

[a]: Grouped according to physical frailty.

[b]: Grouped according to frailty index.

Model 1: Adjusted for age and sex.

Model 2: Adjusted for all covariates.

Appendix Table 11. Association of frailty and RIDs with death as a competing risk, UK Biobank (UK, 2006–2022)

RIDs	Events/all participants	Fully-adjusted Model	
		HR (95% CI)	*P* -value
RIDs			
Non-frail[a]	11389/269882	1.00 (reference)	-
Pre-frail[a]	8917/144734	1.29 (1.26, 1.33)	<0.001
Frail[a]	1002/9075	1.94 (1.82, 2.09)	<0.001
Non-frail[b]	10228/253296	1.00 (reference)	-
Pre-frail[b]	8665/144591	1.48 (1.45, 1.51)	<0.001
Frail[b]	2415/25804	2.20 (2.13, 2.27)	<0.001
per 1-point increase for PF		1.24 (1.22, 1.26)	<0.001
per 0.1-point increase for FI		1.47 (1.45, 1.48)	<0.001
Subgroups of RIDs			
Influenza			
Non-frail[a]	1204/269882	1.00 (reference)	-
Pre-frail[a]	930/144734	1.30 (1.19, 1.42)	<0.001
Frail[a]	131/9075	2.42 (2.01, 2.91)	<0.001
Non-frail[b]	1055/253296	1.00 (reference)	-
Pre-frail[b]	921/144591	1.38 (1.27, 1.52)	<0.001
Frail[b]	289/25804	2.10 (1.84, 2.40)	<0.001
per 1-point increase for PF		1.30 (1.23, 1.36)	<0.001
per 0.1-point increase for FI		1.41 (1.34, 1.49)	<0.001
OA-LRTI			
Non-frail[a]	12903/269882	1.00 (reference)	-
Pre-frail[a]	10162/144734	1.23 (1.20, 1.26)	<0.001

RIDs	Events/all participants	Fully-adjusted Model	
		HR (95% CI)	*P* -value
Frail[a]	1102/9075	1.69 (1.59, 1.81)	<0.001
Non-frail[b]	10455/253296	1.00 (reference)	-
Pre-frail[b]	10529/144591	1.49 (1.45, 1.53)	<0.001
Frail[b]	3183/25804	2.15 (2.06, 2.24)	<0.001
per 1-point increase for PF		1.21 (1.19, 1.23)	<0.001
per 0.1-point increase for FI		1.46 (1.44, 1.49)	<0.001
Pneumonia			
Non-frail[a]	10638/269882	1.00 (reference)	-
Pre-frail[a]	9627/144734	1.39 (1.35, 1.43)	<0.001
Frail[a]	1298/9075	2.27 (2.14, 2.40)	<0.001
Non-frail[b]	8899/253296	1.00 (reference)	-
Pre-frail[b]	9411/144591	1.47 (1.43, 1.51)	<0.001
Frail[b]	3253/25804	2.33 (2.23, 2.43)	<0.001
per 1-point increase for PF		1.33 (1.31, 1.35)	<0.001
per 0.1-point increase for FI		1.51 (1.48, 1.53)	<0.001

Abbreviations: RIDs, Respiratory Infectious Diseases; OA-LRTI, Other acute lower respiratory tract infection; PF, physical frailty; FI, frailty index.

Hazard ratios and 95% CIs were estimated using Fine and Gray competing-risks regression, with death treated as a competing event. A two-sided p < 0.05 was considered statistically significant.

[a]: Grouped according to physical frailty.

[b]: Grouped according to frailty index.

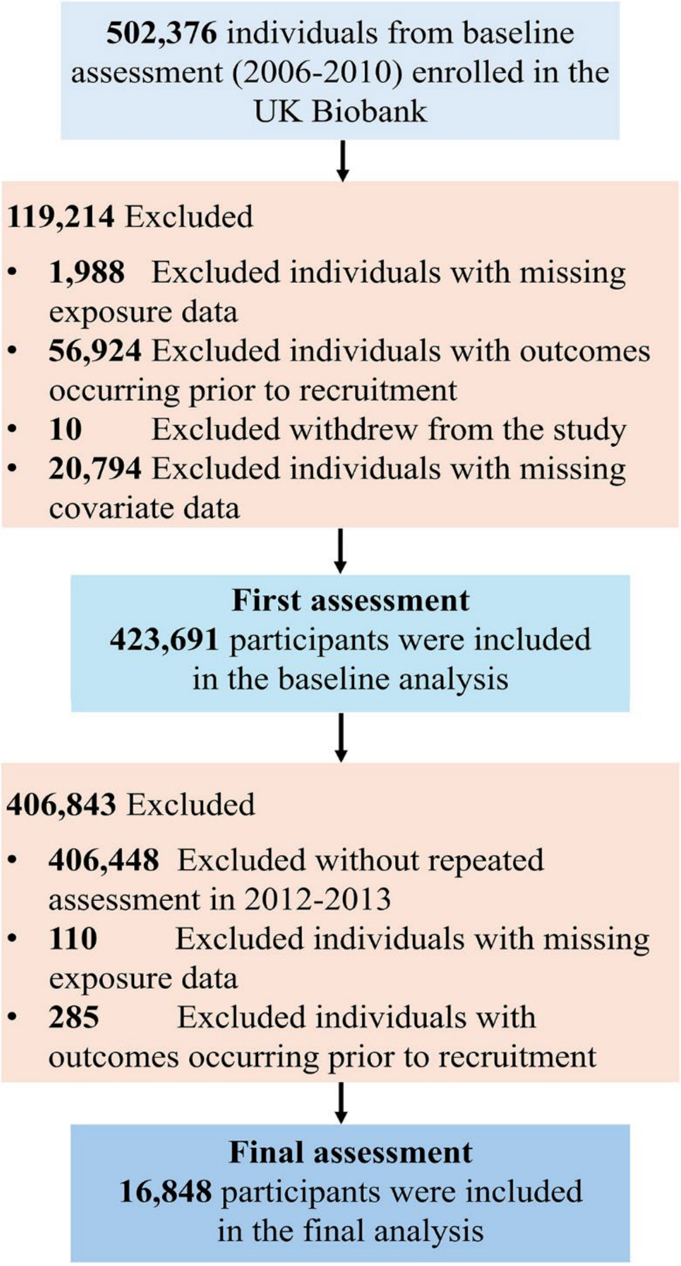

Appendix Figure 1. Flowchart of participants

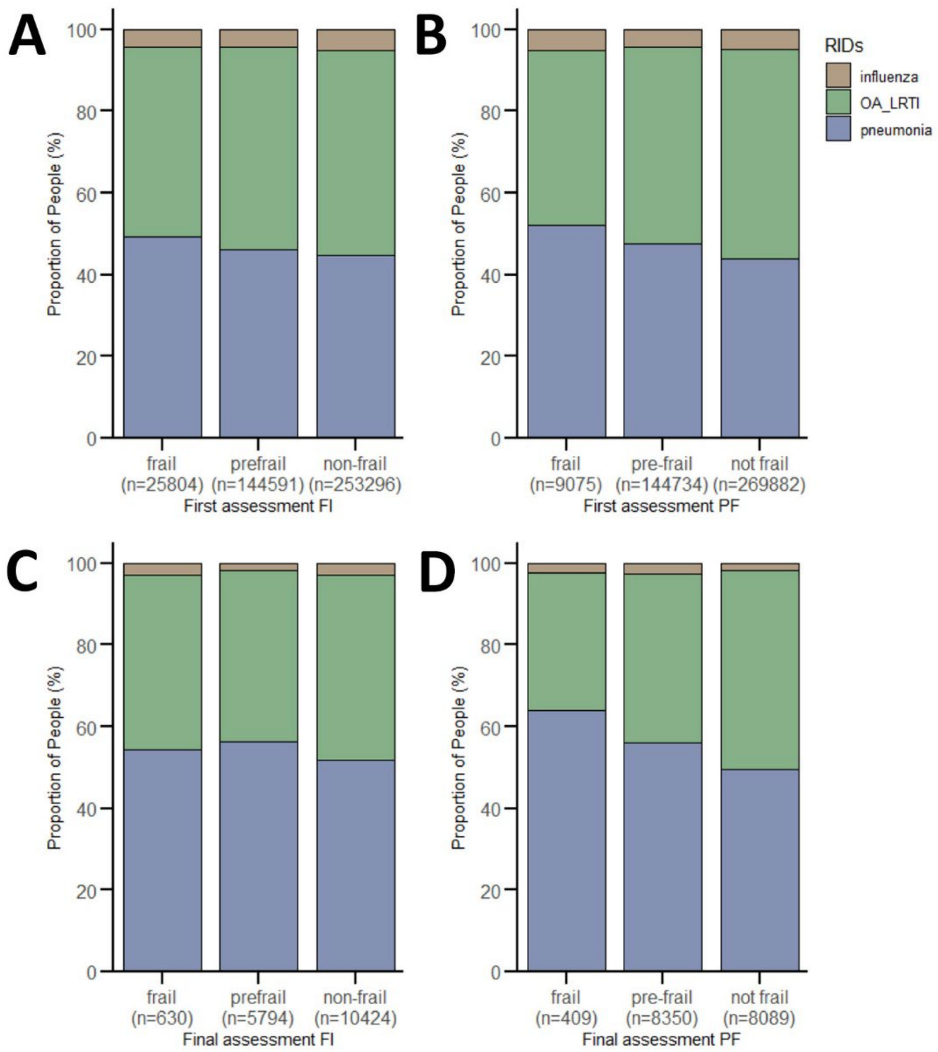

Appendix Figure 2. The distribution of RIDs in the first and final assessments

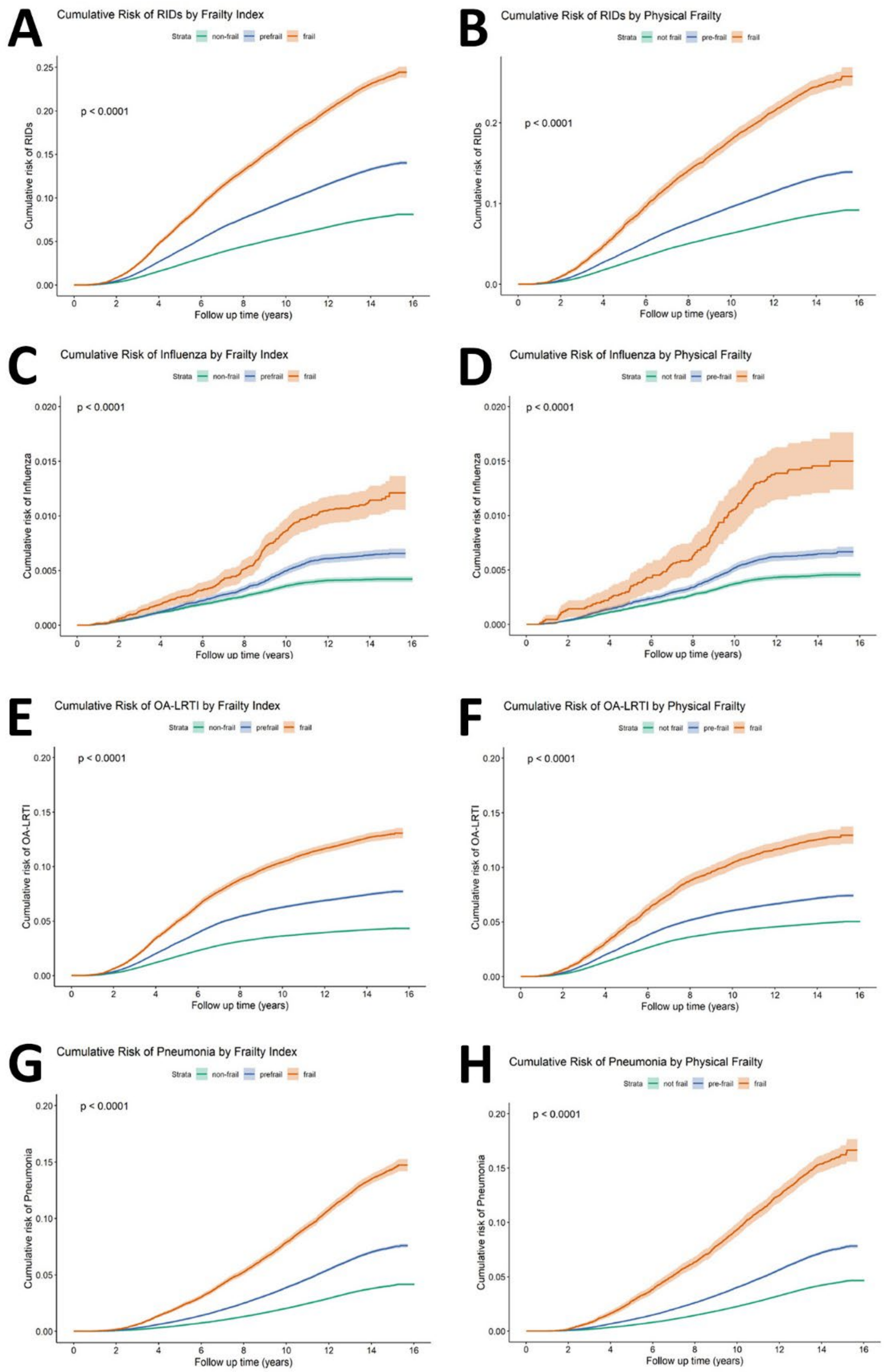

Appendix Figure 3. Cumulative risks of incident RIDs according to frailty in the first assessments

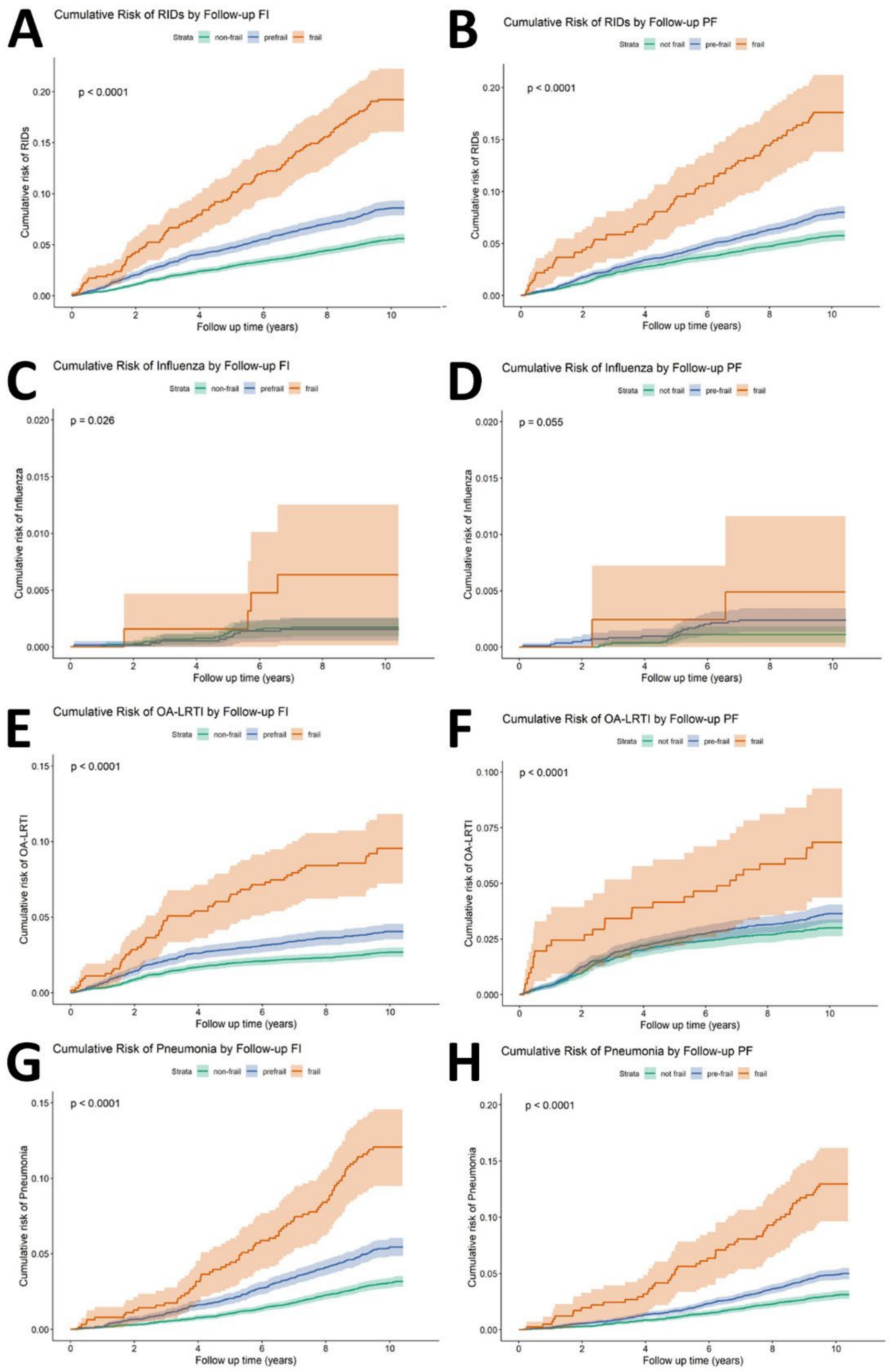

Appendix Figure 4. Cumulative risks of incident RIDs according to frailty in the final assessment

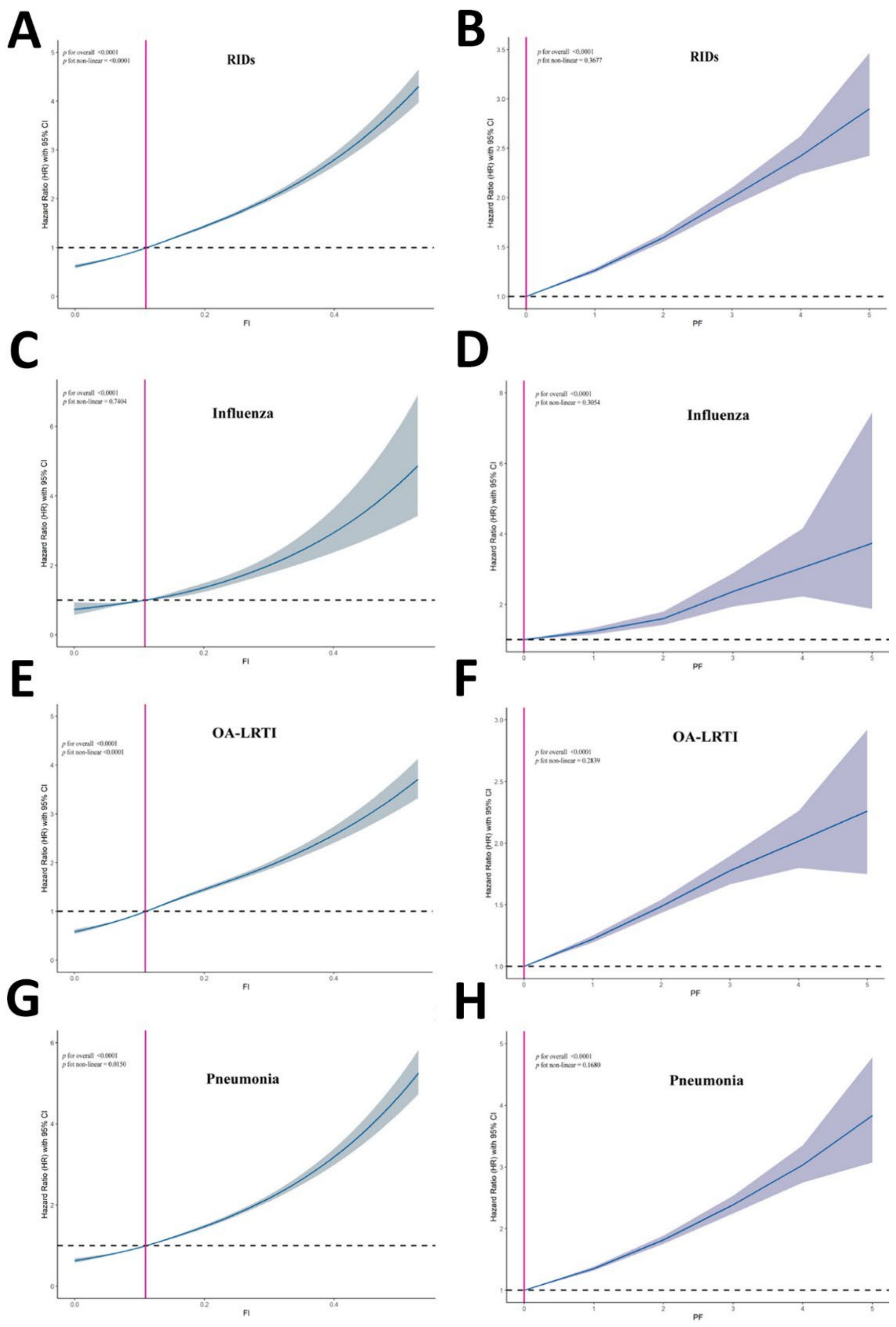

Appendix Figure 5. Dose-response association of frailty with risk of incident RIDs in the first assessment. Dose-response association of frailty with risk of incident RIDs in the first assessment. Panel A, C, E, G represent the first assessment of frailty index associated with RIDs, Influenza, OA-LRTI, and

pneumonia, respectively; Panel B, D, F, H depict the first assessment of physical frailty associated with RIDs, Influenza, OA-LRTI, and pneumonia, respectively. Analyses were adjusted for the following covariates: age; sex; ethnicity; education level; Townsend deprivation index; smoking status; alcohol consumption status; cumulative dietary risk factor score; $PM_{2.5}$; NO_2; nitrogen oxides; BMI category; sleep duration; sleeplessness; daytime dozing. Abbreviations: RIDs, Respiratory Infectious Diseases; OA-LRTI, Other acute lower respiratory tract infection; PF, physical frailty; FI, frailty index.

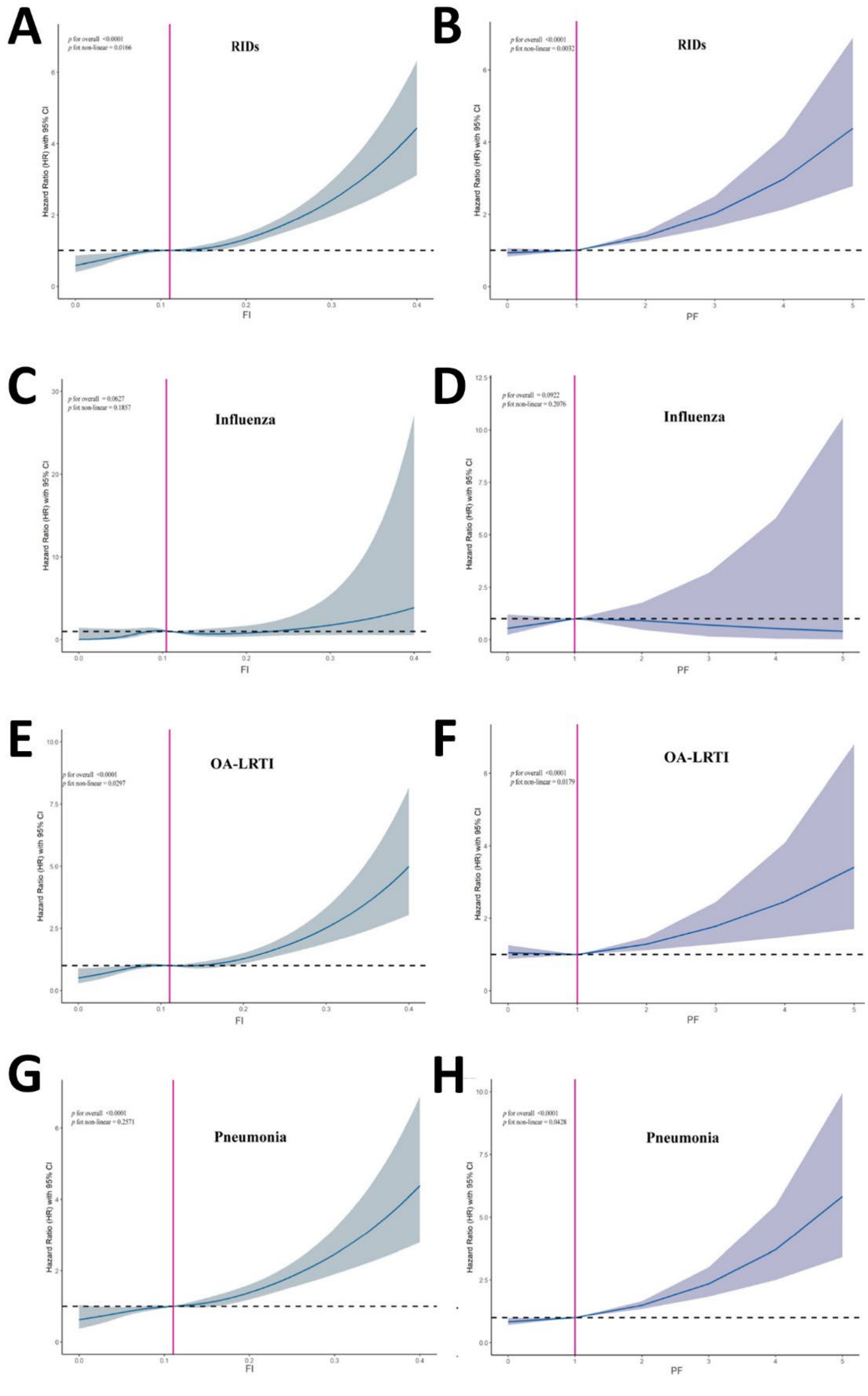

Appendix Figure 6. Dose-response association of frailty with risk of incident RIDs in the final assessment. Dose-response association of frailty with risk of incident RIDs in the final assessment. Panel

A, C, E, G represent the final assessment of frailty index associated with RIDs, Influenza, OA-LRTI, and pneumonia, respectively; Panel B, D, F, H depict the final assessment of physical frailty associated with RIDs, Influenza, OA-LRTI, and pneumonia, respectively. Analyses were adjusted for the following covariates: age; sex; ethnicity; education level; Townsend deprivation index; smoking status; alcohol consumption status; cumulative dietary risk factor score; PM2.5; NO2; nitrogen oxides; BMI category; sleep duration; sleeplessness; daytime dozing. Abbreviations: RIDs, Respiratory Infectious Diseases; OA-LRTI, Other acute lower respiratory tract infection; PF, physical frailty; FI, frailty index.
